# Valvulogenesis of a living, innervated pulmonary root induced by an acellular scaffold

**DOI:** 10.1038/s42003-023-05383-z

**Published:** 2023-10-07

**Authors:** Magdi H. Yacoub, Yuan-Tsan Tseng, Jolanda Kluin, Annemijn Vis, Ulrich Stock, Hassiba Smail, Padmini Sarathchandra, Elena Aikawa, Hussam El-Nashar, Adrian H. Chester, Nairouz Shehata, Mohamed Nagy, Amr El-sawy, Wei Li, Gaetano Burriesci, Jacob Salmonsmith, Soha Romeih, Najma Latif

**Affiliations:** 1 Magdi Yacoub Institute, Harefield, UK; 2 National Heart and Lung Institute, Imperial College London, London, UK; 3 Aswan Heart Science Center, Magdi Yacoub Foundation, Aswan, Egypt; 4 Department of Cardiothoracic Surgery, Erasmus MC, Rotterdam, The Netherlands; 5 Amsterdam UMC, University of Amsterdam, Department of Cardiothoracic Surgery, Amsterdam, The Netherlands; 6 Royal Brompton and Harefield Hospital, London, UK; 7 Cardiovascular Medicine, Brigham and Women’s Hospital, Harvard Medical School, Boston, USA; 8 Department of Bioengineering, Imperial College London, London, UK; 9 Department of Computing, Imperial College London, London, UK; 10 Cardiovascular Engineering Laboratory, UCL Mechanical Engineering, University College London, London, UK; 11 Bioengineering Unit, Ri.MED Foundation, Palermo, Italy

**Keywords:** Cardiac device therapy, Cardiovascular biology, Interventional cardiology

## Abstract

Heart valve disease is a major cause of mortality and morbidity worldwide with no effective medical therapy and no ideal valve substitute emulating the extremely sophisticated functions of a living heart valve. These functions influence survival and quality of life. This has stimulated extensive attempts at tissue engineering “living” heart valves. These attempts utilised combinations of allogeneic/ autologous cells and biological scaffolds with practical, regulatory, and ethical issues. In situ regeneration depends on scaffolds that attract, house and instruct cells and promote connective tissue formation. We describe a surgical, tissue-engineered, anatomically precise, novel off-the-shelf, acellular, synthetic scaffold inducing a rapid process of morphogenesis involving relevant cell types, extracellular matrix, regulatory elements including nerves and humoral components. This process relies on specific material characteristics, design and “morphodynamism”.

## Abstract (web summary)

This study describes a method for in situ recruitment of relevant cells and extracellular matrix using a novel acellular scaffold to produce a living pulmonary root.

## Introduction

Heart valve disease continues to be a major cause of mortality and morbidity worldwide^1^ and there is no effective pharmacological therapy for treating valve disease. Surgical or percutaneous valve replacement are the only available methods for treating patients with advanced heart valve disease. To date there are no ideal heart valve substitutes^2,3^. Native heart valves perform extremely sophisticated functions^4^ which depend on the viability of their components^2^. These functions translate into important end points such as longevity and quality of life^2,5^. This has stimulated extensive attempts at tissue engineering a living heart valve substitute capable of reproducing most, or all of the functions of the living native valve. Most of these attempts relied on the use of different cells in combination with a variety of scaffolds^6–9^ or decellularized scaffolds^10–13^. Using cells and/or scaffolds made of animal origin in tissue engineering has major disadvantages such as precluding off the shelf use, as well as ethical and practical issues. In situ regeneration^14–19^ provides a platform for avoiding the use of exogenous cells, the exact methods of achieving that remains undefined^5,20^, and the mechanisms involved remain unknown. The next generation tissue engineered valves^15,21^ should have repair, remodelling and regeneration capacity emulating the properties of a living valve^2^ and last the lifetime of the patient^21^. We here describe the design of the Heart Biotech Composite Component Valve (HCCV), which is comprised of a multi-layered, novel, jet-sprayed polycaprolactone (PCL) scaffold^22^, that stimulates in vivo valvulogenesis. This is detailed in space and time in an ovine model, and we discuss the mechanisms involved. In addition, we present and discuss in vitro and in vivo functionality of the HCCV.

## Results

The HCCV is a PCL composite component valve geometrically tailored akin to the native human semi-lunar valve.

### Production of the HCCV

**Fig. 1:**
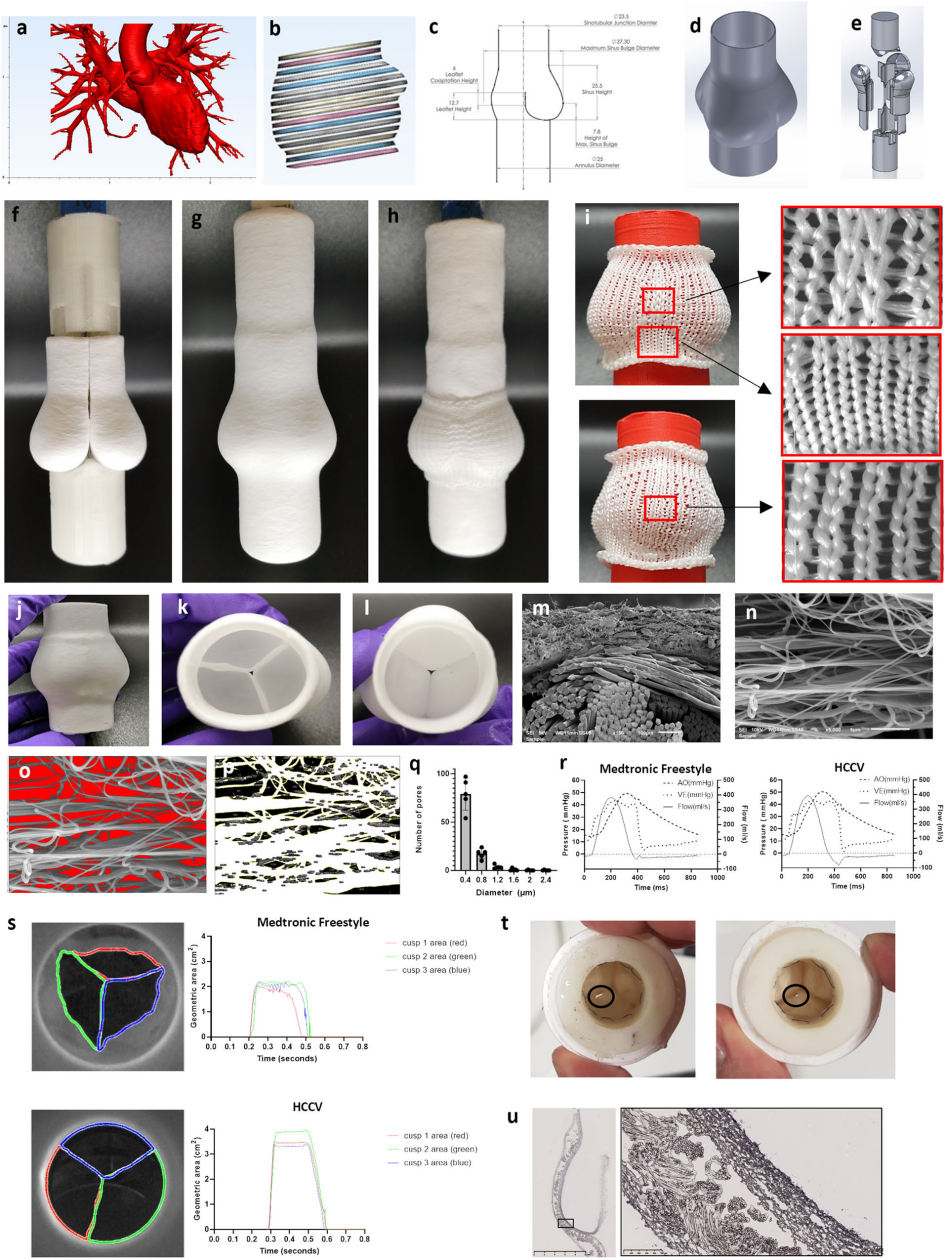
Design and characterisation of the HCCV. A CT scanned image of a normal aortic root used as a template for designing the HCCV **a**; Segmentation of the root region **b**; 2D drawing of the components of the HCCV (all units are in mm) **c**; 3D computer-assisted design (CAD) of the external geometry **d**; valve assembly where one-third of the HCCV was modelled to the shape of a single sinus, leaflet and one third of ascending artery and 3 of these sinus models were assembled to create the complete HCCV **e**. A holder was designed to house the 3 sinus models and to create the ascending artery. A counter mould was designed to complementarily fit the sinuses to create the interleaflet triangles and the annulus **e**. Three jet sprayed sinus moulds and housed into the holder (top) with the counter mould (bottom) **f**. Whole mould assembly jet sprayed **g** with the knitted support **h**. Pictures showing the design of the knitted support with 2 sinus view (top) and one sinus view (bottom) with regions of magnification of the different regions **i**. Final HCCV after the dissolution of the PVA moulds **j** showing top view **k** and annular view **l**. SEM through the wall of the sinus showing the knitted PCL support (bottom fibres) and nanofibers (top) ×150 **m**. SEM of nanofibers x5000 **n**; analysis of the empty spaces between the nanofibers **o**, threshold image **p**, and a histogram of pore size distribution (all data is represented as mean (±SD), *n* = 5, independent samples) **q**. Representative pressure-flow relationships of the Medtronic Freestyle valve and the HCCV **r**. Representative geometric areas of the 3 leaflets **s**. Each leaflet’s opening area was assigned 3 different colours to show their synchronisation between the Medtronic Freestyle valve (top, *n* = 1) and the HCCV (bottom, *n* = 1) **s**. Tears (indicated by black circle) in 2 HCCVs after 9 million cycles in the accelerated wear tester **t**. Cross-section of the HCCV with high magnification of the sinus wall showing the knitted support embedded within nanofibers **u**.

The scaffold consists of a biocompatible, bioresorbable, acellular, porous PCL scaffold with the specific geometry of a human, native semi-lunar valve. This was based on the 3D reconstruction of images of normal aortic roots, obtained from the Aswan Heart Center following ethical approval (Fig. 1a, b). The design was slightly modified to enhance the longitudinal curvature of the sinuses of Valsalva and the height of the leaflets in an attempt to enhance the fluid to solid interaction and improve coaptation^23^ (Fig. 1c). A 25 mm annulus size, computer assisted design (CAD) model was created depicting the external geometry (Fig. 1d) as well as the leaflet surfaces (Fig. 1e). Moulds were 3D printed in water dissolvable polyvinyl alcohol (PVA) of one third of the valve consisting of a combined sinus and leaflet which were customised to fit into a holder and counterbalance (Fig. 1e). Each sinus/leaflet mould was jet sprayed with nanofibrous PCL. The nanofibers were jet sprayed to produce an anisotropic scaffold with fibres predominantly in the circumferential direction. Three sinus/leaflet moulds were embedded into a holder (Fig. 1f) and this assembly was jet sprayed (Fig. 1g). A knitted support (patent EP 3 552 577 B1) with proximal and distal sewing rings was placed on top of the sinuses (Fig. 1h) and this was jet sprayed again. The knitted PCL support was created by warp knitting in a jersey design using PCL yarn to allow expansion in the circumferential direction (Fig. 1i). The PVA moulds were removed by dissolving in water to reveal the HCCV (Fig. 1j–l). The sinus walls consisted of a knitted support embedded within jet-sprayed nanofibers of PCL (Fig. 1m) and the leaflets consisted of jet-sprayed nanofibers only. SEM was used to characterise the nanofiber size (mean 0.38 µm (±0.02)) and representative images of the PCL nanofibrous scaffold (Fig. 1n–p) showed the pore diameter to be of mean size of 0.32 µm (±0.03) with median of 0.24 µm (±0.04) (Fig. 1q) and of varying shapes (Fig. 1o).

### In vitro testing

Hydrodynamic testing of HCCV scaffold (*n* = 4) and the Medtronic Freestyle 25 mm (*n* = 4) showed adequate effective orifice area of 2.15 cm^2^ (±0.06) and 2.04 cm^2^ (±0.41) respectively, *p* = ns; trivial to mild pulmonary regurgitation, 11.47% (±0.58) and 4.56% (±1.28) respectively, *p* = 0.0001; closing volumes of −7.02 ml (±1.75) and −0.82 ml (±0.23) respectively, *p* = 0.0004; leakage volumes of −4.47 ml (±2.05) and −3.73 ml (±1.07), *p* = ns (Table 1, Fig. 1r). The peak systolic pressure gradient of HCCV and Medtronic Freestyle was 23.37 mmHg (±1.11) and 23.46 mmHg (±3.43) respectively, *p* = ns. Instantaneous movement of the leaflets showed a high degree of synchrony between the 3 leaflets during opening (Fig. 1s). The leaflet kinetics as determined by a high-speed camera (Supplementary video 2 and 3) showed the geometric orifice area of HCCV and Medtronic Freestyle valve was 3.13 cm^2^ (±0.14) and 2.21 cm^2^ (±0.14) respectively, *p* = 0.0001. The duration of opening of the HCCV and Medtronic Freestyle valve leaflets was 0.325 s (±0.014) and 0.302 s (±0.018) respectively, *p* = ns. The opening rate of the HCCV and Freestyle valve leaflets was 74.15 cm^2^/s (±13.00) and 56.73 cm^2^/s (±11.42) respectively, *p* = ns. The closing rate of the HCCV and Medtronic Freestyle valve leaflets was −29.54 cm^2^/s (±5.92) and −22.99 cm^2^/s (±2.72) respectively, *p* = ns. Durability testing of the unpopulated HCCV demonstrated continued functionality up to 9 million cycles, which equates to a period of 3 months, at which time point small tears were observed in the belly region (Fig. 1t). Microstructure of the HCCV scaffold showed layering of the nanofibers and the macrofibrous knitted support (Fig. 1u).

**Table 1:** Summary of in vitro hydrodynamic performance of HCCV and control (Medtronic Freestyle Valve).

	HCCV (*n* = 4)	Medtronic Freestyle (*n* = 4)	Significance	*P* value
Effective orifice area (cm^2^)	2.15 (±0.06)	2.04 (±0.41)	no	0.5994
Regurgitation fraction (%)	11.47 (±0.58)	4.56 (±1.28)	yes	0.0001
Closing volume (ml)	−7.02 (±1.75)	−0.82 (±0.23)	yes	0.0004
Leakage Volume (ml)	−4.47 (±2.05)	−3.73 (±1.07)	no	0.5490
Peak systolic pressure gradient (mmHg)	23.37 (±1.11)	23.46 (±3.43)	no	0.9606
Geometric orifice area (cm^2^)	3.13 (±0.14)	2.21 (±0.14)	yes	0.0001
Duration of opening (s)	0.325 (±0.014)	0.302 (±0.018)	no	0.0864
Opening rate (cm^2^/s)	74.15 (±13.00)	56.73 (±11.42)	no	0.0908
Closing rate (cm^2^/s)	−29.54 (±5.92)	−22.99 (±2.72)	no	0.0910

All data are represented as mean (±SD) with *n* = 4 independent samples.

### In vivo preclinical testing

Following insertion of the HCCV in the pulmonary position, a rapid program of repopulation is initiated, simultaneously from the luminal and from the adventitial side. HCCV were explanted at intervals of 1 week (*n* = 1), 2 weeks (*n* = 1), 3 weeks (*n* = 1), 9 weeks (*n* = 1), 12 (*n* = 7), and 26 weeks (*n* = 4) (Supplementary Fig. 1).

**Fig. 2:**
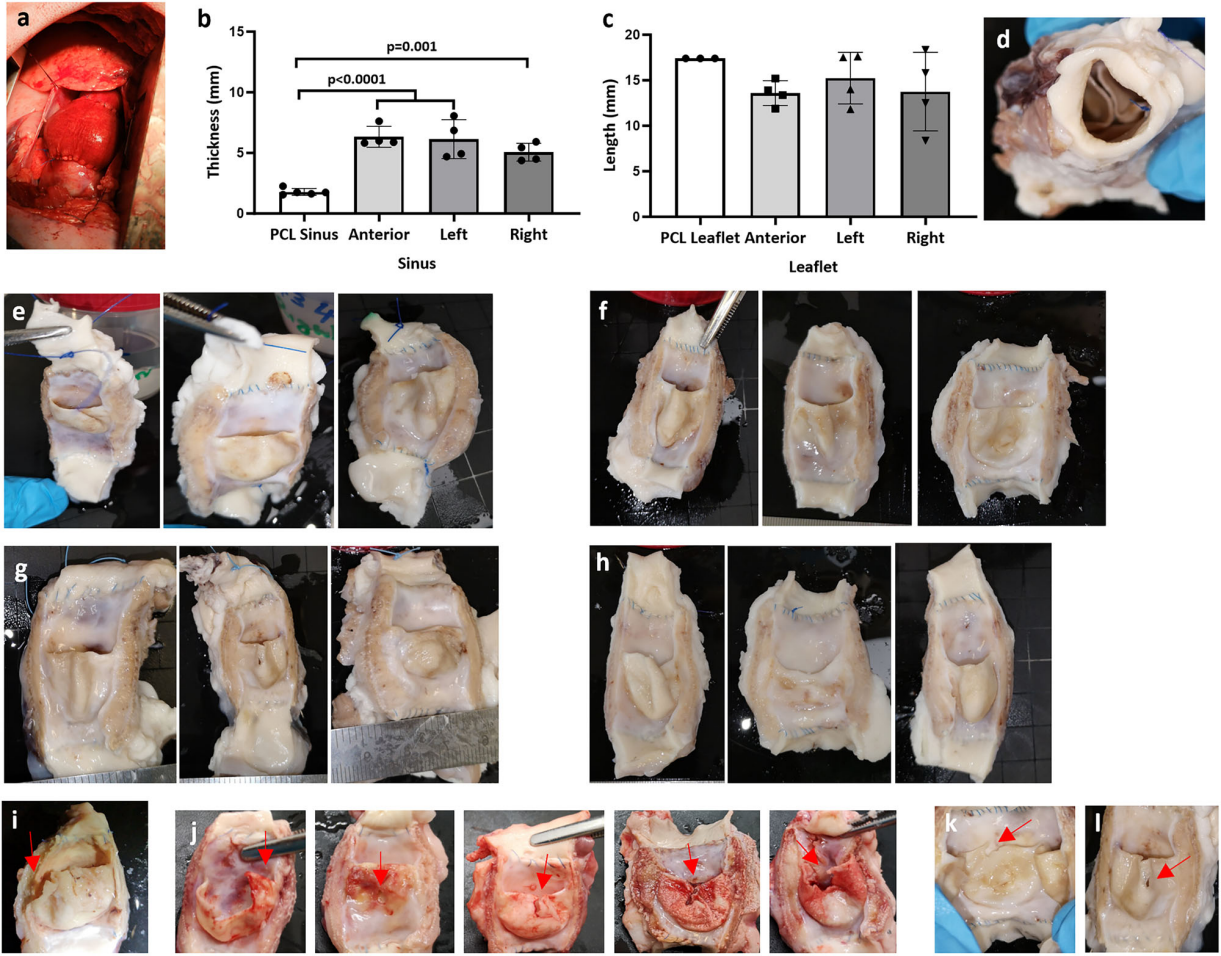
Macroscopic images of explanted HCCV with increases in sinus wall thickness. The functional implanted view of HCCV immediately after implantation **a**. Graphs showing mean sinus thicknesses **b**, and mean leaflet lengths **c** at 26 weeks post-implantation, *n* = 4 (mean ± SD) biologically independent samples) using one-way ANOVA followed by Tukey’s multiple comparison test. The arterial view of HCCV at 1 week post-implantation **d**. Open views of 4 HCCV at 26 weeks post-implantation ordered anterior, right posterior, and left posterior for each HCCV **e**–**h**. Leaflets showing the position of tears at 9 weeks **i**, 12 weeks **j**, **k**, and 26 weeks **l** post-implantation.

On implantation, the HCCV showed good visible function with no leakages on anastomotic rings (Supplementary Video 1) (Fig. 2a). At 26 weeks post-implantation, the sinus walls showed a significant increase in the thickness from 1.75 mm (±0.27) pre-insertion to 6.76 mm (±1.28) (*p* < 0.0001), 5.35 mm (±1.70) (*p* < 0.0001) and 5.03 mm (±2.02) (*p* = 0.0001) post insertion for the anterior, left and right sinuses respectively (Fig. 2b). All leaflets at 6 months post-implantation underwent a minor, non-significant degree of retraction from the original height of 17.40 mm (±0.00): the anterior retracted to a mean height of 13.60 mm, (±1.37 mm), the left facing leaflet retracted to mean of 15.20 mm, (±2.83 mm) and the right facing leaflets retracted to height of 13.80 mm, (±4.31 mm) (Fig. 2c). There was a significant increase in leaflet thickness from a mean of 0.62 mm (±0.02 mm) pre-implant to a mean of 1.25 mm (±0.82 mm), at 6 months post-implantation, *p* < 0.0001. On explantation, gross appearance of the HCCV, at all the time points, showed no signs of calcification or thrombi (Fig. 2d–l). The surfaces were smooth, and the shape of the sinuses was preserved. The leaflets were thin, pliable with shiny surfaces and good coaptation (2–4 mm). 8 of the 15 HCCV had small tears with 1 HCCV at 9 weeks (1 leaflet), 5 HCCV at 12 weeks (7 leaflets) and 2 HCCV at 26 weeks (2 leaflets) (2–3 mm in length) in the cusps originating from the coapting edge (Fig. 2i–l). On further analysis, the 9 week explant showed evidence of infection in the leaflets but not the wall.

### Timing and phenotype of repopulating cells and ECM production

**Fig. 3:**
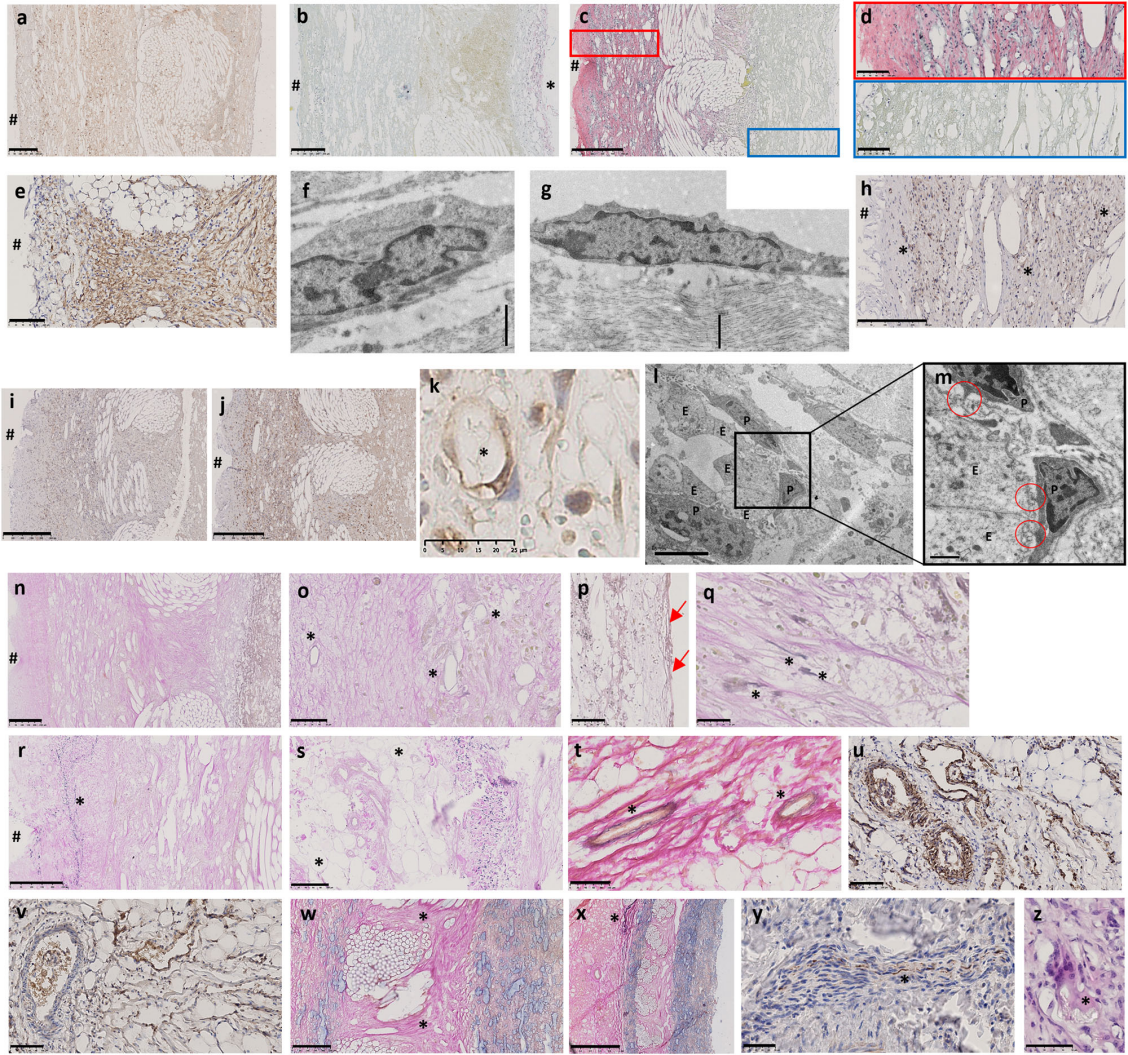
1, 2, and 3 weeks post-implantation sections of HCCV. 1-week post-implantation of HCCV showing representative sinus wall images for CD45 immunostaining **a**, collagen by EVG (*) on luminal side **b**; 2 weeks post-implantation (ABSR and haematoxylin staining) **c**, magnified boxes in **c** **d**. Cells expressing α-SMA **e**, TEM showing fibroblast **f** and myofibroblast **g** morphology, ECs (CD31) lining neo-vessels (*) **h**, M1 macrophage expression **i**, M2 macrophage expression **j**, neo-vessel (*) and adjacent pericyte **k**, TEM showing pericytes adjacent to ECs **l**, **m** with peg/socket connections (red circles). Collagen **n** and elastogenesis in neo-vessels (*) **o**, neo-intima (arrow indicating elastin) **p**, neo-media **q**, incomplete capsule (*) **r**, adipogenesis in the neo-adventitia (*) **s** by EVG. 3 weeks post-implantation showing lamellae in neo-vessels (EVG) **t**, α-SMA **u**, and SMM **v** by immunostaining, collagen EVG **w** in the knitted region, elastin sheath on the outer knit (EVG) **x**, nerve bundle stained with neurofilament protein **y**, and FBGCs (*) **z**. # Indicates adventitial side. Scale bars: 250 µm on **a**, **b**, **h**, **m**, **q**, **v**; 500 µm in **c**, **I**; 100 µm in **d**, **e**, **r**, **s**, **t**, **u**; 1 µm in **f**, **g**, **l**; 5 µm in **k**; 25 µm in **j**, **p**; 50 µm in **n**, **o**, **x**, **y** and 1mmm in **w**. E represents ECs and P represents pericyte.

At 1-week explantation, the earliest cells to appear within the sinus wall were bone marrow derived CD45-positive cells (pan leukocyte marker) in greater numbers from the adventitial side (Fig. 3a) with a few cells penetrating into the centre of the scaffold and within the knitted support (see Supplementary Table 1 for antibody information). In addition, there were a few myofibroblasts stained positive with α-SMA with early deposition of collagen (Fig. 3b) in patchy areas on the luminal side only.

By week 2, the sinus wall remodelled into 2 layers consisting of a neo-media incorporating the knitted support, and a neo-adventitia with concomitant collagen deposition (Fig. 3c). There was a greater abundance of populating cells, particularly on the neo-adventitia (Fig. 3c) compared to the luminal side (Fig. 3d). The populating cells were of different phenotypes (CD45 cells (pan leukocyte marker), myofibroblasts (SMA-positive) (Fig. 3e, f), fibroblasts (Fig. 3g), endothelial cells (CD31) (Fig. 3h), and classically activated pro-inflammatory M1 (CCR7) (Fig. 3i), and alternative activated anti-inflammatory M2 (CD163) macrophages (Fig. 3j). CD163 was more abundantly expressed than CCR7. Scarce multinucleated foreign body giant cells (FBGCs) were also detected. Neoangiogenesis was observed at this early stage, with a single endothelial lining present (Fig. 3h, k). These were seen within the neo-adventitia up to the luminal side of the knitted support. Importantly, these neo-vessels showed the presence of peripheral pericytes through peg and socket connections to endothelial cells (ECs) on their abluminal sides (Fig. 3k–m). The appearance of adipogenesis was first observed at the proximal and distal edges of the HCCV within the neo-adventitia, harbouring neo-vessels (Fig. 3e, s).

At this stage, collagen fibres were detected predominantly in a longitudinal direction on both sides of the knitted support with longitudinal fibres extending across the knitted support (Fig. 3n). Elastogenesis in the form of fine elastin fibres, was observed in the neo-vessels (Fig. 3o), neo-intima (Fig. 3p), neo-media (Fig. 3q), and in the beginning of an incomplete sheath in the outer neo-adventitia (Fig. 3r).

The 3-week explant was characterised by further development of the arterial wall and the vasa vasorum with the beginnings of lamellae (Fig. 3t) in the media and adventitia containing smooth muscle cells (SMCs) strongly expressing α-SMA (Fig. 3u), and slightly weaker expression of smooth muscle myosin (SMM) (Fig. 3v). Most of the cells in the neo-media stained positive for α-SMA and SMM. Further deposition of collagen on either side of the knitted support by fibroblasts and myofibroblasts was observed particularly on the adventitial side with thicker bridges of collagen across the knitted support (Fig. 3w, x). This was accompanied by further elastogenesis in the form of a thin sheath (Fig. 3x) in the outer knit simulating the external elastic lamina.

A new and exciting feature was the appearance of a small group of unmyelinated nerve bundles (300–400 µm diameter) weakly expressing the neural marker neurofilament protein (Fig. 3y) formed in situ in the newly formed adipose tissue adjacent to neo-vessels. Multinucleated FBGCs (Fig. 3z) as well as M1 and M2 macrophages increased in numbers in the adventitia and in the knitted support.

**Fig. 4:**
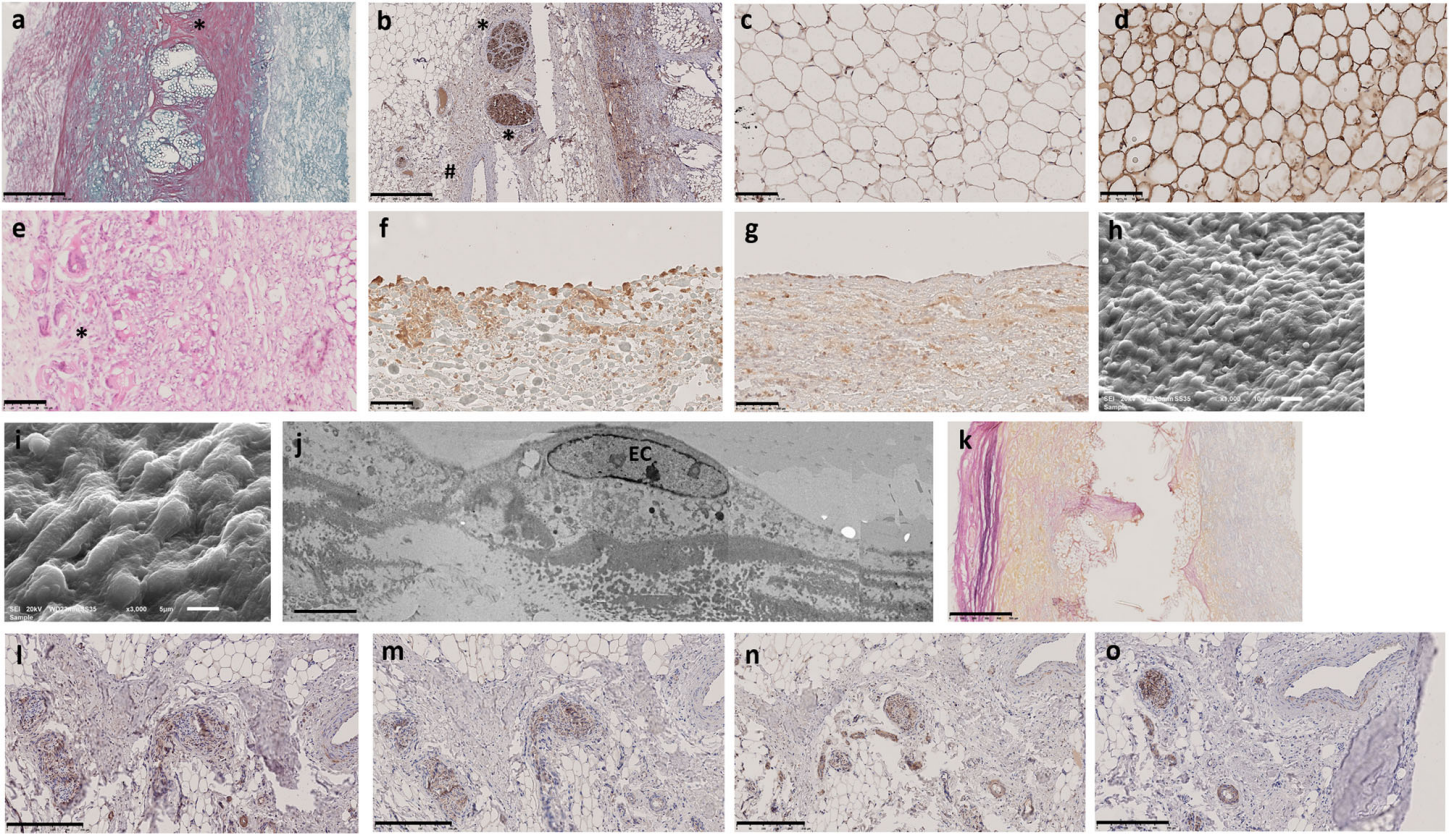
9 and 12 weeks post-implantation sections of HCCV wall. HCCV showing collagen deposition stained by EVG **a**, nerve bundles immunostained stained with neurofilament protein **b**, embedded within expanding adipose tissue stained with FABP4 on the adventitial side **c** at 9 weeks post-implantation and leptin **d** at 12 weeks post-implantation. Complete cellularisation of the sinus wall stained with H&E showing a layer of multicellular FBGCs (*) **e**, cuboidal ECs on the sinus wall **f**, longitudinal ECs on the arterial side of the leaflets **g** stained with CD31, SEM of ECs on the sinus wall **h**, **i**, TEM of EC **j** at 9 weeks post-implantation, incomplete elastin capsule stained with EVG **k** at 9 weeks post-implantation, nerve bundles stained with VIP **l**, TH **m**, NPY **n**, and neurofilament protein **o** at 12 weeks post-implantation. Scale bars: 500 µm in **a**, **b** and **k**; 250 µm in **l**, **m**, **n**, **o**; 100 µm in **c**, **d**, **e**, **f**, **g**; 10 µm on panel **h**; 5 µm in **i** and 0.5 µm in **j**.

The 9-week explant showed enhanced collagen synthesis in the knitted support and outer adventitia (Fig. 4a). Expansion of nerve bundles stained with neurofilament protein (Fig. 4b) in number (5–12) and size (40 µm–1.3 mm diameter) was observed in the outer sinus adjacent to large vessels and within adipose tissue stained with FABP4 (Fig. 4c) and leptin (Fig. 4d). The sinus wall was completely cellularised (Fig. 4e) with a combination of fibroblasts, myofibroblasts (staining positive for α-SMA) and macrophages M1, M2 and a layer of FBGCs lined the outer neo-media (Fig. 4e). A patchy layer of cuboidal endothelial cells (ECs) expressing vWF on the surface of the sinus wall (Fig. 4f) and occasional longitudinal ECs on the leaflets (Fig. 4g) were demonstrated for the first time. Importantly these cells had the morphology (Fig. 4h, i) and ultrastructure of normal ECs (Fig. 4j). On the external surface of the adventitia of one sinus wall there was an incomplete sheath consisting of collagen and elastic fibres (Fig. 4k). All the repopulating cells and ECM appeared to originate from the circulation and the surrounding tissues at anastomotic sites.

The 7 explants at 12 weeks post-implantation showed continuing remodelling and strengthening by collagen. There were many nerve bundles ( ~ 20) in the sinuses of all 7 HCCV which stained positive for vasointestinal peptide (VIP) (Fig. 4l), tyrosine hydroxylase (TH) (Fig. 4m), neuropeptide Y (NPY) (Fig. 4n) and neurofilament protein (NF) (Fig. 4o). VIP and NPY also stained some of the SMCs of the vasculature (Fig. 4l, n). The scaffold was populated by FBGC throughout the wall of the sinuses and up to the hinge region. A continuous layer of ECs was observed on the sinus walls which lay above a fibrous layer consisting of myofibroblasts and fibroblasts. Most of the arterial sides of the leaflets were covered in ECs (Fig. 4g).

**Fig. 5:**
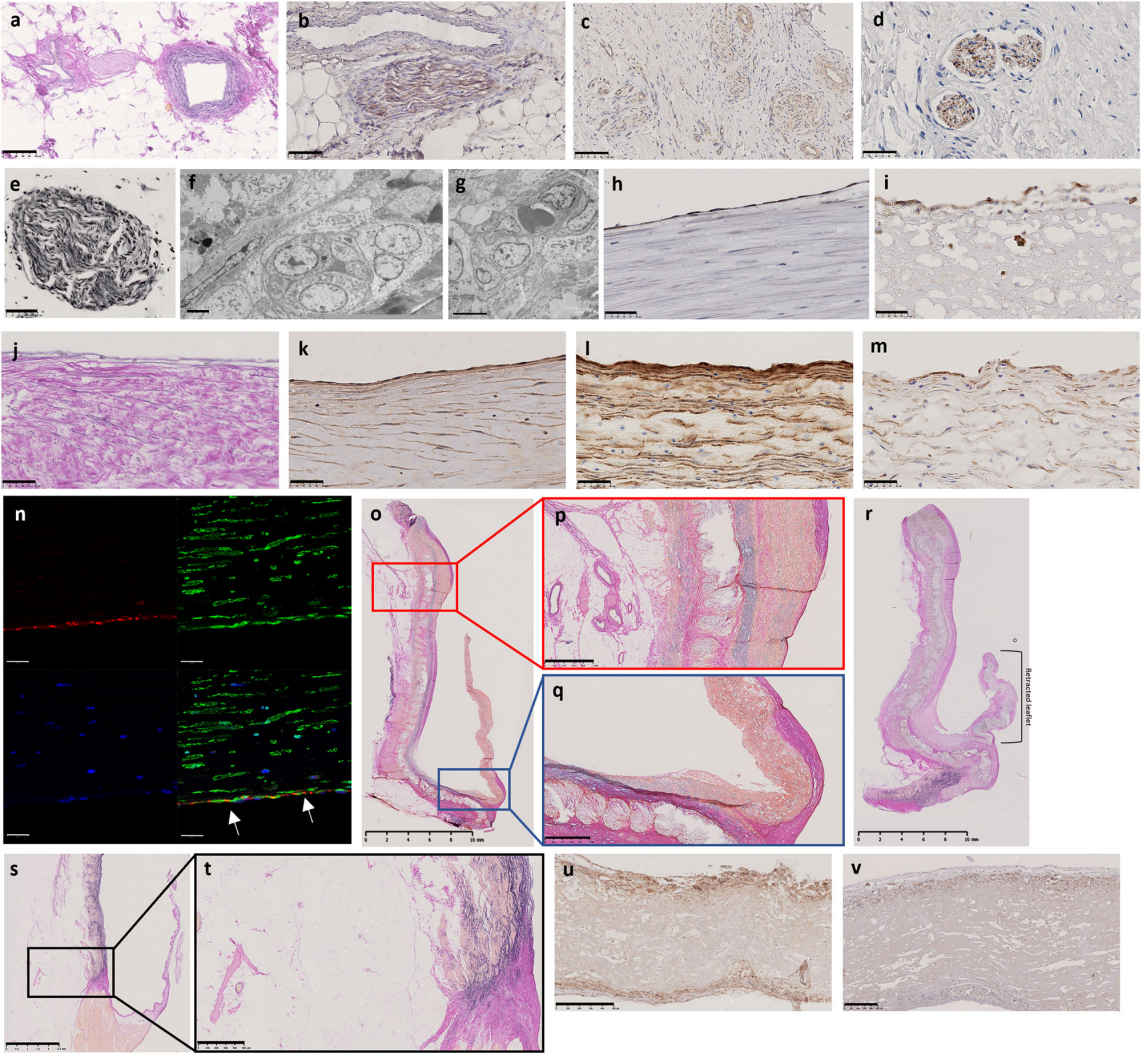
26 weeks post-implantation sections of HCCV. HCCV showing maturation of the vessels residing in the adventitial adipose layer adjacent to a nerve bundle stained with EVG **a**; tyrosine hydroxylase immunostained nerve bundle **b**, neuropeptide Y **c**, neurofilament protein **d**, silver stained nerve bundle **e**, ultrastructure of nerve bundles by TEM **f**, **g**, VwF immunostaining on sinus wall **h**, arterial side of leaflet **i**, EVG stained EC basement membrane **j**, elastin immunostaining **k**, α-SMA **l**, SMM **m**, co-expression of CD31 (red) and α-SMA (green) **n**, full root section showing adipose tissue, elastin and collagen staining by EVG **o**, magnified panels showing adipose tissue with nerve bundles and vessels **p**, collagen creeping on ventricular aspect of leaflet **q**. Retracted leaflet **r**, normal pulmonary root **s** showing adipose tissue housing a nerve bundle and vessels in close proximity with magnified image **t**. HCCV leaflet immunostained with α-SMA **u**, CD163 **v**. Upper side in **u** and **v** is leaflets’ arterial side. Scale bars: 100 µm in **a**–**c**; 50 µm in **d**, **e**, **h**, **i**, **j**, **k**, **l**, **m**; 2 µm in **f**; 5 µm in **g**; 20 µm in **n**; 10 µm in **o**; 1 mm in **p**, **q**; 2.5 mm in **s**; 500 µm in **u** and 250 µm in **v**.

By 26 weeks there was further maturation of all the layers of the sinus wall and the cellular and ECM components, strongly supporting the arterial wall by interlacing collagen fibres, elastic tissue, and SMCs. At this stage, there was a very well-developed adventitial layer containing the regulatory elements such as nerves, vasa vasorum, and white adipose tissue (Fig. 5a). 3 of the 4 explants at 26 weeks showed the presence of many nerve bundles positive for tyrosine hydroxylase (Fig. 5b), neuropeptide Y (Fig. 5c), and neurofilament protein (Fig. 5d) to varying degrees in each bundle, as well as positive staining for silver, which are markers of mature neural tissue (Fig. 5e). Ultrastructure of axons and Schwann cells, was also confirmed by electron microscopy (Fig. 5f, g).

A continuous endothelial lining stained with vWF (Fig. 5h) was formed on the sinus wall with a well-defined intimal layer and an internal elastic lamina (Fig. 5j). The endothelial lining (vWF) on the leaflets was nearly complete (Fig. 5i). ECs and intima expressed elastin (Fig. 5k), α-SMA (Fig. 5l), and SMM (Fig. 5m) on the sinuses (similar to a normal sheep sinus wall (Supplementary Fig 2)). ECs on the luminal side of the sinus wall and microvessels showed endothelial-to-mesenchymal transformation (EndMT) by the co-expression of the EC marker (CD31) and mesenchymal marker α-SMA (Fig. 5n). Fibroblasts, detected by their morphology and ultrastructure using transmission electron microscopy (Fig. 5g), and myofibroblasts, detected using α-SMA without the presence of SMM were found throughout the sinus wall including in the fibrotic layer, which is underneath the sinus endothelium, either side of the scaffold, within the knitted layer, the neo-media and adventitia. There was an increased amount of adipose tissue (Fig. 5o, p), similar to a normal pulmonary sinus wall (Fig. 5s, t). This contained white adipocytes housing neurons and vessels (Fig. 5o, p). The hinge region was supported by a layer of collagen located along the ventricularis (Fig. 5p, q). Of the 4 HCCV explanted at 26 weeks, only one leaflet out of 12 showed retraction (Fig. 5r).

In the leaflets, there was no collagen, elastin, or proteoglycan deposition. Cells were predominantly of the myofibroblastic (Fig. 5u), fibroblastic, endothelial, M2 (Fig. 5v) and FBGC phenotype, in decreasing incidence. The hinge region follows a slow process of recellularization similar to that observed in the leaflets.

### Valve function and adaptation

**Fig. 6:**
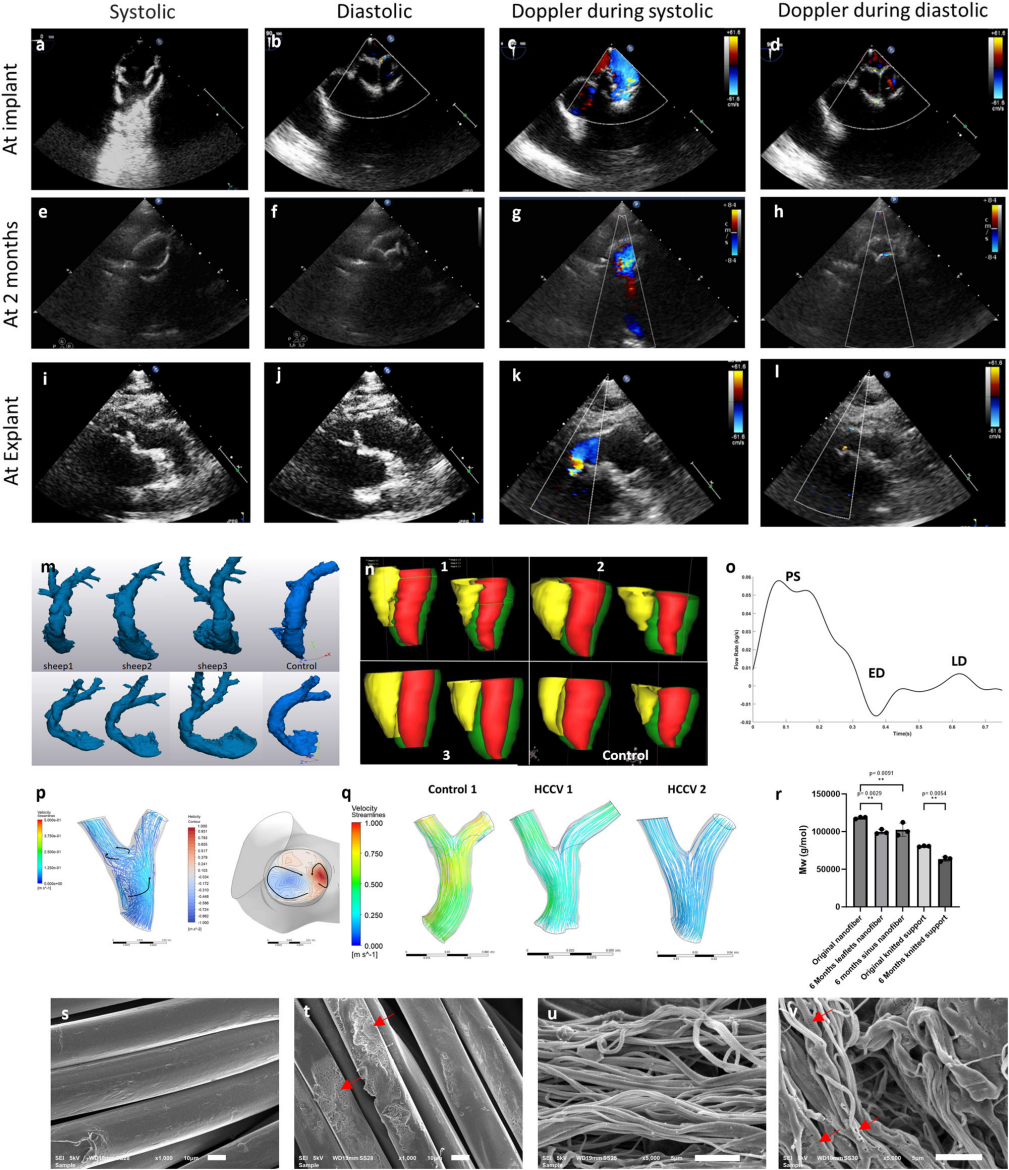
In vivo function and degradation of the HCCV. First row shows the echocardiograms of the HCCV at implant during systole **a** and diastole **b**, followed by Doppler ultrasound showing laminar flow during systole **c** and minimal regurgitation during diastole **d**. The second row shows the HCCV at 2 months during systole **e** and diastole **f**, followed by Doppler ultrasound during systole **g**, and diastole **h**. Third row shows the HCCV at 6 months during systole **i** and diastole **j**, followed by Doppler ultrasound showing systole **k** and trace regurgitation during diastole **l**. MRI generated root geometry of 3 HCCVs and one control at 26 weeks post-implantation **m**. MRI generated ventriculogram showing right (yellow) and left (red) ventricles in 3 HCCV and one control **n**, instantaneous flow of HCCV at annulus (PS, peak systole; LD, late diastole: ED, early diastole) **o**, CFD showing the pattern of flow through the pulmonary artery bifurcation of one HCCV and 2 opposing vortices **p**, CFD showing parallel velocity streamlines in one control (normal sheep) and through 2 HCCVs at peak systole at 6 months post-implantation **q**. Molecular weight of original and 26 weeks implanted PCL nanofibers in leaflets, sinuses, and knitted support (all data is represented with mean ± SD, *n* = 3, (biologically independent samples) **r**, SEM analysis of knitted fibres before **s** and after **t** implantation at ×1000 (red arrows indicating erosion), SEM of nanofibers before **u** and after **v** implantation at ×5000 (red arrow indicating “pitting”). Scale bars are 5 µm in **s** and **t**; 10 µm in **u** and **v**.

In vivo echocardiography of the HCCV showed good movement and coaptation of the leaflets with minimal regurgitation (Fig. 6a–l, supplementary Fig. 4). MRI-generated geometry and function showed preserved root shape with pronounced sinuses (Fig. 6m), preserved size and function of the right ventricle (Fig. 6n) with conserved end systolic (ES) and end-diastolic (ED) volumes. There was no evidence of stenosis or regurgitation of the HCCV. There was an excellent size match between the HCCV and the right ventricular outflow tract (Fig. 6m). Instantaneous flow determination from MRI showed near normal tracings (Fig. 6o). Computational Fluid Dynamics (CFD) showed parallel streamlines in the main pulmonary artery during the systolic phase and double helix formation in the main branches during diastole (Fig. 6p), while showing no abnormal acceleration similar to the normal sheep (control), (Fig. 6q). Strain analysis of the HCCV roots was conducted using MRI at the pulmonary sinus’s maximum bulge during the cardiac cycle. The strain measured at 6 months for HCCVs (*n* = 5) was 0.42 (±0.16) compared to 0.51 (±0.08) in native sheep (*n* = 3), although there appears to be a decrease in strain for the HCCV root, this difference is not statistically significant.

### Scaffold degradation

Gel permeation chromatography showed significant PCL nanofiber degradation at 6 months from original PCL nanofiber with an average MW 118374 Da (±1349) to an average MW 99734 Da (±3070) for nanofibers within leaflets (*p* = 0.0029) and an average MW 102573 Da (±8726) for sinus walls (*p* = 0.0091) (Fig. 6r). There was no significant difference in MWs between samples tested in the leaflets to those tested in the sinuses after 6 months of implantation. The PCL knitted support showed slow degradation at 6 months from the original support with an average MW 80554 Da (±708) to an average MW 63489 Da (±2983), *p* = 0.0054 (Fig. 6r). SEM analysis of the knitted fibres showed degradation of their smooth surface (Fig. 6s, t) while the nanofibers showed some “pitting” after 6 months’ implantation (Fig. 6u, v).

### Incidence of calcification

Of the 14 HCCV explanted, only 1 leaflet showed one very small nodule of calcification at 6 months (500 µm in diameter) which did not impact valve function (Supplementary Fig. 3a–c). There were no signs of calcification in any of the sinuses.

## Discussion

This manuscript shows for the first time that following the insertion of an acellular scaffold of specific multi-layered design^22,24^, there is an active program of valve morphogenesis resulting in a living and functioning valve. The design takes advantage of solid to fluid interaction^23^, morphodynamism^4,23^, stress sharing between the sinuses and the leaflets^25^, and the specific size and shape of the pores in the scaffold^26,27^.

In vitro functionality of the HCCV scaffold showed adequate effective orifice area and peak systolic pressure gradient compared to the Medtronic Freestyle. Regurgitation fraction of the HCCV was below the ISO standard requirement of 15% however it was significantly higher than the Medtronic Freestyle, mainly contributed by the increasing closing volume of the HCCV. This is likely contributed by the significantly higher geometric orifice area of the HCCV. However, following repopulation of the scaffold, in vivo studies showed trace regurgitation which did not affect ventricular volume. In vitro durability testing of the HCCV scaffold showed functionality up to 9 million cycles, (equivalent to a period of 3 months of normal cardiac cycles) at which time point tears appeared. However, in vivo, the HCCV surpassed this with good, functional performance for up to 6 months due to the remodelling capability of the scaffold. Similarly, the tritube decellularized valve exceeded the in vitro number of cycles to failure^28^. Functionality of the HCCV was also maintained as supported by the echo results with no signs of stenosis or significant regurgitation. Furthermore, MRI showed preserved size and shape of the ventricles demonstrating the good functionality of the HCCV throughout the time course of implantation.

The remodelling program is associated with the recruitment and differentiation of cells to the relevant phenotype as in the native valve such as SMCs, myofibroblasts, fibroblasts and ECs^29–31^ with the production and layering of the extracellular matrix components. The layering in the HCCV leaflets was not as mature as in normal pulmonary leaflets however the layering in the HCCV sinus walls was comparable to that of a normal pulmonary sinus wall with defined intimal, medial, and adventitial layers^32,33^. Valve morphogenesis is an extremely complex process^34^ involving genetic, mechanical and molecular pathways with interactions of multiple cell types. The spatiotemporal process of morphogenesis induced by the scaffold is similar to but not identical to that during valve development^34,35^. This includes interactions at the cell surface, between fibroblasts, activated myofibroblasts, pericytes, ECs, immune cells, SMCs, and ECM^27,36^. We observed activated myofibroblasts at 2 weeks after insertion, which increased in number over time, particularly in the neo-adventitial layer followed by the neo-media and neo-intima. These cells were involved in producing collagen, elastin and other elements of the ECM. ECs were detected on the scaffold’s surface as well as in the neo-vessels at 2 weeks and showed a cobble-stone appearance expressing CD31 by 9 weeks. By 12 weeks there was an almost complete EC layer on the sinus walls. Pericytes were observed adjacent to the ECs of the neo-vessels. ECs and pericytes in the neo-vessels are thought to play a vital role in angiogenesis and adipogenesis^37,38^. Pericytes are known to influence the development and function of ECs in arterial walls^39^. Evidence of EndMT was first seen at 9 weeks in the intimal ECs. EndMT plays a major role in valvulogenesis by repopulating leaflets with new cells influencing valve homeostasis in the long term^34,40^. The newly formed vessels were closely associated with nerve bundles. The cellular composition of the HCCV wall approached that of the native sinus wall by 6 months with ECs on the surface, SMCs in the media, myofibroblasts, fibroblasts, adipocytes, and neurons in the adventitia. However, macrophages and foreign body giant cells persisted at the 6-month time point. Macrophages were shown to be the “pioneering “ cells in the remodelling of a decellularized porcine pulmonary valve^12^. The foreign body response and wound healing are the most hypothesised mechanism for the integration and remodelling of the scaffold^17,41,42^.

An important unique feature is the development of neurogenic tissue at 3 weeks and increasing progressively over time. These nerves expressed several neurotransmitters, which could play an important role in reducing the inflammatory response^43^. The presence of nerves has not been documented in any other tissue engineered valve. Nerves play an important regulatory role in the development, maturation and normal physiology of valves^44,45^ and nerve fibres have been shown to originate from the adventitial wall^46^. In situ neurogenesis by an acellular, porous scaffold has been shown but this was in a model of nerve injury^47^.

White adipose tissue was first observed in the outer neo-adventitia at 3 weeks after the development of angiogenesis^48^. This adipose tissue may contribute to unique mechanical behaviour of the sinus wall acting as a “cushion” similar to that observed in the normal pulmonary sinus wall. Adipose tissue has many regulatory and metabolic functions, which could be of relevance to morphogenesis and function of the mature valve^49^ and has not been documented in any other tissue engineered valve. The development of the adventitia in tissue engineered valves is poorly documented but may be the master regulator of wall physiology and pathobiology with functional and structural roles^50^. The neural network in the adventitia releases adrenergic, cholinergic, peptidergic, purinergic and nitrergic neurotransmitters that lead to SMC contraction or relaxation^50^. Identification of these in the HCCV is ongoing with limited availability of sheep specific antibodies. The adventitia is also a source of resident stem and progenitor cells for cell renewal and differentiation^50,51^.

The development of multiple layers in the sinus wall was initiated by week 3, and by week 9 included an intimal layer containing elastic fibres and fibroblasts, a neo-media containing myofibroblast-like cells and elastic fibres and a circumferential fibrous layer around the internal support connected with collagen bridges. This functioned as the main support to the sinus wall. The neo-adventitia began to appear by 3 weeks progressively increasing in size. This is known to play a major regulatory role in the function of vessels^52^. This early remodelling of the HCCV is favourable as the scaffold begins to degrade from the point of implantation. In contrast, early intimal coverage of the XPV and Gen 1 valves was not documented as the studies’ earliest time point was 2 and 3 months respectively^18,28^.

The process of morphogenesis was advanced in the sinuses by 26 weeks and this was attributed to the design characteristics, particularly the inclusion of the knitted support in the sinuses. Repopulation of the leaflets and hinge mechanism was significantly slower which is similar to the Xeltis, and Gen 1 and 2 valves^18,28^. However, Kluin et al. have shown abundant circulating cells throughout the microporous structure of their valve by 2 months^53^.

The diverse population of the cells repopulating the scaffold originates from circulating cells. They enter the scaffold from the adventitial surface and the luminal side, and the former appears to be the dominant route. The neo-vasculature may act as an additional entry site of cells, and the pannus extending over the HCCV also allows for cell recruitment from a nearby tissue. Future studies are needed to identify routes for cell recruitment into neo-leaflets.

All these processes resulted in a highly functional valve with rapid opening and no evidence of obstruction or regurgitation. The response of the right ventricle and the pattern of flow in the distal artery reflected the sophisticated function of a living valve. Sequential echos as well as MRIs have shown preserved sinus shape of the HCCV, conserved right ventricular size and pattern of flow when compared to normal sheep as well as lack of stenosis. The current study is the first to use post implantation sequential MRI to yield both anatomical and functional data related to the HCCV.

Retraction of the leaflets has been problematic in tissue engineered valves however this was observed in only 1 out of 12 leaflets in our study, comparatively lower than in other studies. To anticipate/circumvent this issue of retraction, the HCCV has been designed with a longer height mid leaflet to maintain coaptation. Other tissue engineered valves have also shown mild and severe infolding in some cases to the degree where the leaflet had fused to the sinus wall^18,54^, shrinkage in all leaflets^10,55^ and a significant reduction in mean cusp length^54,56^.

The nanofibrous PCL and macrofibrous PCL yarn both showed a similar level of degradation as assessed by GPC suggesting that hydrolytic degradation is the principal mode of breakdown and not cellular enzymatic breakdown as the sinuses were highly populated with cells compared to the leaflets. In contrast, the Xeltis valve showed more advanced absorption/degradation in the conduit as compared to the leaflets^18^ based on the use of different polymers with different degradation rates in each location. The stented valve produced by Kluin et al. showed a modest decrease in molecular weight of all fibres with the disappearance of the polymer after 12 months in cell rich regions^53^.

A very small nodule of calcification was seen in only 1 leaflet out of 4 HCCVs at 6 months and in none of the sinuses. This is a similar result to the Xeltis valve with leaflet calcification in 1 leaflet of 5 valves^18^ and in a tri-tube valve which showed punctate microcalcification in some leaflet regions^28^. Kluin et al. showed that only sparse spots were present in two 12-month explants^53^. The sheep model is particularly prone to calcification, so our result is a favourable outcome.

Study limitations include the small number of animals in early time points, relatively short period of follow-up and the lack of experiments demonstrating growth capability. The slower repopulation of the leaflets requires further investigation and is similar to other valves^12,28^. The observation of small tears in 7 of the 15 HCCVs is another limitation though tears in 1 explant were due to an infection; however, these tears were extremely small and surrounded by living tissue, which could limit extension and may stimulate healing. These tears occurred at points of high stress, at the coapting edges and commissures and risk of tearing would be decreased by slightly thickening the leaflets.

Novel findings in the remodelling of the HCCV show early elastogenesis in the sinuses with adipogenesis on the outer sinus wall to include angiogenic and neurogenic systems. The documentation of repopulation of the HCCV with appropriate cells and ECM, both structurally and functionally, is extremely promising and should translate to clinically relevant results, both in terms of quality of life and longevity, when used in man.

## Methods

### Creation and manufacturing of HCCV valve

The creation and manufacture of the HCCV involved a multi-step process. First, 3D modelling of the pulmonary root was based on images of normal aortic roots provided by the Aswan Heart centre. The images were segmented using Mimics (Materialise NV, Belgium,) and used to create moulds in Solidworks (Dassault Systèmes). The design was slightly modified to extend the longitudinal curvature of the sinuses of Valsalva and the height of the leaflets in an attempt to enhance the fluid to solid interaction and improve coaptation^23^ (Fig. 1c). A 25 mm annulus size, computer assisted design (CAD) model was created depicting the external geometry (Fig. 1d) as well as the leaflet surfaces (Fig. 1e). Each cusp was modelled separately with a counterbalance that is removable to permit high speed rotation. A holder and a counter mould were designed to hold the 3 cusps together with the help of a Y-struct to mimic the shape of the root. The Individual moulds were 3D printed in polyvinyl alcohol (PVA) using a 3D printer (Ultimaker 3) except the Y-struct which was CNC machined in titanium for mechanical support (Protolab) (Fig. 1e). Second, jet spraying of nanofibrous scaffold: This process has been described previously and modified to adapt to the multi-part moulds spraying process^22^. Briefly, compressed air passed through a custom-made jet sprayer while the 10% w/v polycaprolactone (PCL) solution is extruded at 35 ml/hour using a syringe pump (Alaris Guardrails Plus). The Jet sprayer was operated at 7 bar pressures through a stainless-steel conical nozzle size of 500 µm in diameter and a needle size of 120 µm in diameter. The jet sprayer is mounted on a XYZ stage to spray the PCL nanofiber onto the moulds at 30 cm distance. Each sinus mould was mounted in a rotating mandrel and rotated at 10000 rpm with an estimate of 12.38 m/s surface speed for the cusp mould to form aligned nanofibers. All 3 cusps were sprayed separately then mounted into the holder as shown in Fig. 1e, f. The assembled mould was jet sprayed at 500 rpm with average surface speed at 0.65 m/s to form the first non-aligned outer layer. The thickness of the nanofibrous layers was measured using a non-contacting confocal thickness profiler (Micro-epsilon, UK) with 10 measurement points per model. Third, creation of knitted support structure: Stoll M1 plus knitting software was used to design different regions of the support structure with different tensions to withstand the pulmonary pressure and support suture retention (patent EP 3 552 577 B1). PCL yarn, 220 dtex (EMS-Griltech), is used to warp knit a specific pattern with regional tensions to conform to the shape and stiffness of the normal valve, while allow expansion in the circumferential direction (Fig. 1i) on a Stoll flat-bed knitting machine (Stoll, UK). The knitted support with proximal and distal sewing rings was placed on top of the sinuses (Fig. 1h) over the sprayed assembled valve and then jet sprayed again at 500 rpm as described earlier. Lastly, the solvent welding process: Ethyl acetate was used to weld the PCL fibres between the outer sinus layers of the sinus moulds and the complete outer layer over the whole 3 sinus moulds. The process was optimised to provide good adhesion between the PCL layers while maintaining porosity for cell penetration. This was achieved through 30 min of solvent welding with 15 ml of ethyl acetate at 30 °C using a custom build 2 L air-tight chamber. The mould with the outer sprayed layer was placed in an air-tight chamber that contained a temperature probe and placed on a rotator for homogenous distribution of the ethyl acetate vapour. This ensured saturation of the chamber and uniform solvent welding. Completed valves were placed in a desiccator under vacuum for 48 h to remove any residual solvent. After residual solvent removal, the jet sprayed valve was immersed in a water bath at 45 °C for the PVA moulds to dissolve. As the moulds dissolve, the Y-strut was removed to prevent damage to the cusps. Once completely dissolved, the valve was dried in a laminar flow cabinet after exchanging with 100% ethanol for 3 times at 1-h intervals. The dried valves were sent for ethylene oxide sterilisation at 35 °C (Test Ltd, UK) prior to in vivo implementation.

The resulting scaffold consists of a biocompatible, bioresorbable, acellular, porous polycaprolactone (PCL) polymer with the specific geometry of a human, native semi-lunar valve (Fig. 1j–l). The sinus walls consisted of a knitted support embedded within jet-sprayed nanofibers of PCL (Fig. 1m) and the leaflets consisted of jet-sprayed nanofibers only (Fig. 1n).

### Scanning electron microscopy

Jet sprayed nanofibers and knitted support were mounted to aluminium sample holders with carbon tape and sputter coated with gold/palladium (Emitech K550X, Quorum Technologies Ltd, Kent, UK). Images were taken on JEOL JSM 6010LA analytical scanning microscope. Acquired images were analysed using Image J.

### In vitro Hydrodynamic testing

The hydrodynamic performance of the HCCV was evaluated using a pulse duplicator (Vivitro Superpump System SP3891, Vivitro, Victoria, BC, Canada). Physiologically equivalent pulmonary pressures and flows were replicated in accordance with the ISO 5840-2:2021 requirements. The valve was mounted on the aortic test chamber and tested at cardiac outputs (COs) of 7 L/min, 2 L compliance chamber, a viscoelastic impedance adapter with source and output compliance set as 10 ml and 40 ml, a mean arterial pressure of 20 mmHg, a fixed heart rate of 70 beats per minute, and systole occupying 35% of the cardiac cycle. Saline at 37 °C was used as testing fluid. The ViViTest software was used to record stable mean arterial pressure, cardiac output flow, measurements of atrial, ventricular, aortic pressures and aortic flow were over 10 consecutive cardiac cycles. The total regurgitant fraction represents the regurgitant volume expressed as a percentage of the stroke volume^57^. Effective orifice area (EOA), which represents the minimal cross-sectional area of the downstream jet emerging from aortic valve orifice, was derived from the continuity equation, applying Gorlin’s formula^58^. Percentage of geometric orifice area were captured using a high-speed camera at 480FPS (Sony, UK), followed by in house Matlab (Mathworks) code for image analysis^59^.

### Accelerated wear test

The functional durability of the valve was evaluated in an in vitro accelerated wear test (AWT) setup, using a VDT3600i AWTsystem (BDC Laboratories, CO, USA). The valve was mounted on a test chamber, with 37 ± 1 °C buffered saline with 1 g/l of sodium azide testing fluid as a fungicide and bactericide, running at 5 Hz cycle rate, set to maintain a peak differential pressure above 40 mmHg across the closed valve for at least 5% of each cycle. The system software provides continual monitoring of the real-time differential pressures, recording only the cycles where pressure conditions complied with the specified testing requirements. The test was set to run until failure. The valve was visually inspected for any signs of damage on a daily basis, and functionally evaluated in the pulse duplicator before and after completing every 5 million cycles.

### In vivo testing

Two ovine model studies were carried out at Mfd Diagnostics GmbH (Wendelsheim, Germany) (Ethical approval of protocol G18-15-067 from the Landesuntersuchungsamt Rheinland-Pfalz, Germany) from an independent laboratory and local German authorities as applicable and at a Dutch facility following ethical approval. One ovine model study was carried out in the Netherlands. Approval for the animal studies was obtained by the Amsterdam University Medical Centers Animal Care Ethics Committee (AVD1180020197705) and are in agreement with the current Dutch law on animal experiments (WOD). To date, 24 sheep have undergone pulmonary valve replacement using the HCCV (Supplementary Fig. 2) and sheep were randomised for follow-up duration. The valve was explanted at different intervals post-surgery, 1 week (*n* = 1), 2 weeks (*n* = 1), 3 weeks (*n* = 1), 9 weeks (*n* = 1), 12 weeks (*n* = 7) and 26 weeks (*n* = 4). 24 female Swifter sheep (average weight, 50 ± 5 kg, average age 1 year) underwent replacement of the pulmonary valve and main pulmonary artery with the 25 mm HCCV. For implantation in Germany, anaesthesia was induced with 2 mg/kg ketamine, 0.02 mg/kg atropine, and an intravenous bolus infusion of 2 mg/kg propofol. Anaesthesia was maintained by the use of continuous propofol. The heart was exposed by a left anterolateral thoracotomy entering the chest through the 4th intercostal space. Systemic anticoagulation was induced with 400 IU of heparin per kilogram. By means of femoral arterial and right atrial venous cannulation, normothermic cardiopulmonary bypass was established. On bypass, 0.01 mg/kg fentanyl and 0.02 mg/kg pancuronium were administered to ensure anaesthesia. With the heart beating, the pulmonary artery was transected, and a segment of the main pulmonary artery and all 3 native leaflets were removed. The valve conduit was implanted by using running 5-0 monofilament sutures (Prolene, Ethicon, Inc). Heparin was reversed with 300 IU of protamine per kilogram after weaning from bypass. The thoracic wall was closed in layers by using resorbable sutures, and an intercostal nerve block with 0.25% bupivacaine was administered. No further anticoagulation was given. All animals received 1000 mg of cefazolin (Apothecon) for the first postoperative week on a daily basis. For pain control, intramuscular buprenorphine injections were given for the first 3 days and thereafter as necessary. All animals received humane care in compliance with the “Guide for the Care and Use of Laboratory Animals” published by the National Institutes of Health (National Institutes of Health publication No. 85-23, revised 1985). Echocardiograph was carried out at 4 and 8 weeks post implant.

In the Netherlands, all the animals came from a local farm and were bred for education purposes. Their general health was checked upon arrival. They were placed in procedural acclimatisation for at least 14 days prior to the experiment. We conducted a detailed animal welfare assessment once a week, during which all animals were thoroughly checked for any clinical symptoms or discomfort. All animals were socially housed in groups of 2 to 10 animals, on wheat straw bedding (Nijssen Fourages, Nieuw-Vennep, the Netherlands). Animals were fed sheep feed pellets (250 gr per day; Kasper Faunafood, Woerden, the Netherlands) and had ad libitum access to meadow hay (Nijssen Fourages, Nieuw-Vennep, the Netherlands) and tap water. The sheep were housed indoors in an air-conditioned room with a temperature ranging between 15 and 21 °C and with 12 h:12 h light:dark cycle. For implantation the following protocol was followed: Antibiotic treatment was started 2 days prior to surgery and continued until 5 days after surgery with Procaine benzylpenicillin/dihydrostreptomycin (1 ml/20 kg IM, Depomycine, 200.000 IE/200 mg/ml, MSD Animal Health, Kenilworth, USA). A buprenorphine patch (5 mcg/h patch; BuTrans, Mundipharma, Cambridge, UK) was taped to the shaved, ventral side of the proximal part of the tail one day prior to the aortic valve surgery to provide preemptive analgesia. On the day of surgery, ketamin/hydrochlorin (10 mg/kg IM; Narketan 100 mg/ml, Vétoquinol, Paris, France) and midazolam (0,4 mg/kg IM; Midazolam Actavis 5 mg/ml, Actavis, New Jersey, US) were administered as preanesthetic medication. Propofol was used to induce (2 to 4 mg/kg IV; Pro¬pofol 20 mg/mL, Fresenius Kabi, Bad Homburg, Germany) and maintain anaesthesia (20 mg/kg/h IV) during surgery. Sufentanil (5 μg/kg IV; Sufentanil-Hameln, 50 mcg/ml, Hameln, Gloucester, UK) was given as pain relief during surgery. A single dose of amiodarone hydrochloride (300 mg IV, Corda¬rone 50 mg/mL, Sanofi, Paris, France) was added to the saline infusion bag before starting cardiopulmonary bypass (CPB). Pain relief consisted of a buprenorphine patch (5 mcg/hr patch, BuTrans, Mundipharma, Cambridge, UK) 1 day before surgery until day 7 after surgery, flunixinemeglumine (0,02 ml/kg IM, Niglumine, 50 mg/ml, Dopharma, Raamsdonksveer, Netherlands) on day 1 after surgery until day 3 and meloxicam (1 mg/kg orally, Metacam, 20 mg/ml, Boehringer Ingelheim, Ingelheim am Rhein, Germany) from the first day after surgery until day 30. As anticoagulation, Nadroparin (3800 IE IM, Fraxiparine, 9,500 IE/ml, Aspen, Durban, South Africa) was given from day 1 until day 30 after surgery. Furosemide (20 mg IM, Centrafarm, 20 mg/2 ml, Furosemide, Etten Leur, Netherlands) was given as diuretic medication between day 1 and day 7. Depomycine was given as antibiotics for the first post-operative week on a daily basis. Ultrasound measurements were taken directly after implantation, at 2 months and 1 day prior to explantation.

### Explantation

While continuing the administration of maintenance anaesthesia during MRI scanning, the animals were transported to the operation theatre after MRI scanning, after which the valve explant procedure was initiated. The pressure gradient over the pulmonary valve was measured invasively in the right ventricle and pulmonary artery after which the animals were exsanguinated under full anaesthesia. Thereafter, the heart valve prosthesis was carefully explanted and stored in formaldehyde 10% for 24 h before transfer to PBS. Samples of the liver, lungs, spleen, kidneys, left ventricle, and right ventricle were collected and stored in formaldehyde 10% for 24 h before transfer to PBS.

### Cardiac magnetic resonance (CMR) acquisition

On the day of explant, all sheep underwent cardiac MRI scanning on a 1.5 T Ingenia scanner (Philips, Netherlands) under full anaesthesia (MRI technicians Anke Wassink and Raschel van Luijk, the Netherlands). Additionally, three extra sheep that did not undergo heart valve implantation underwent MRI scanning in similar fashion, to serve as healthy controls. On the day of MRI scanning, all animals received ketamin/hydrochlorin (10 mg/kg IM; Narketan 100 mg/ml, Vétoquinol, Paris, France) and midazolam (0,4 mg/kg IM; Midazolam Actavis 5 mg/ml, Actavis, New Jersey, US) as a pre-anaesthetic. Propofol was used as induction anaesthesia (2–4 mg/kg IV; Propofol 20 mg/ml, Fresenius Kabi, Bad Homburg, Germany) and as maintenance anaesthesia (4–7 mg/kg IV; Propofol 20 mg/ml, Fresenius Kabi, Bad Homburg, Germany) in addition to Sufentanil (5 μg/kg IV; Sufentanil-Hameln, 50 mcg/ml, Hameln, Gloucester, UK).

Systolic and diastolic volumes were assessed using a retrospective electrocardiogram-gated steady-state free precession sequence. Vertical long-axis 2- and 4-chamber views and short-axis views consisting of 12 to 14 contiguous slices were acquired, covering both ventricles from the base of the heart to the apex. Scan parameters were; repetition time = 3.2 to 3.8 ms; echo time = 1.6 to 1.9 ms; flip angle = 50–70o; slice thickness = 6–8 mm without slice gap; matrix = 160 × 256; field of view = 350–400 mm, and temporal resolution approximately 25 ms.

2D CMR Flow dynamics across the pulmonary artery were assessed using a retrospective electrocardiogram-gated velocity-encoded MRI sequence. The sequence was encoded for a through-plane velocity 150 cm/s or higher according to the degree of the main pulmonary artery. Scan parameters were repetition time = 9 ms, echo time = 5 ms, flip angle = 15–20o, slice thickness = 6–8 mm, matrix = 128 × 256, temporal resolution approximately 20 ms.

### CMR post-processing analysis

#### Ventricular volumes and function

Biventricular systolic function and 2D flow were analysed with the software CVI 42 (Circle Cardiovascular Imaging, Calgary, Canada) Biventricular systolic function was assessed by drawing endocardial LV and RV contours at end-diastole and end-systole in all sections of the cine short-axis data. End-diastolic volumes (EDV), and end-systolic volumes (ESV) were obtained and indexed for body surface area according to the Mosteller formula: (√ Height (cm) × weight (kg)/3600). Ventricular stroke volume indexed for body surface area was calculated by subtracting ESV from EDV. The ejection fraction was calculated by dividing stroke volume by EDV.

#### 2D CMR flow

Vascular contours were drawn for the pulmonary artery to generate flow-versus-time curves throughout the cardiac cycle. Forward flow, backward flow, and net flow were measured. Cardiac output (CO) (net pulmonary flow) was measured as the product of net flow and heart rate. Cardiac index (CI) (pulmonary flow) was CO indexed to body surface area (BSA).

#### Computational fluid dynamics (CFD)

CFD was performed to visualise flow patterns, “vortex” helical development through the main PA, LPA, and RPA through colour-coded streamlines, vector velocities, the vorticity and helicity contour plots using Fluent (ANSYS Inc., Canonsburg, PA, USA). 3D reconstruction (segmentation) of the main PA, LPA, and RPA was performed using Mimics (Materialise NV, Belgium) to define the domain of the CFD. The fluid domain was defined as blood with a density of 1060 kg/m^3^ and viscosity of 0.0035 Pa s. The boundary conditions were defined using an inlet boundary condition of velocity data at the valve level, and pressure outlet boundary conditions at the LPA and RPA. The inlet velocity data was derived from 2D CMR flow rate data at the valve level.

#### Strain analysis

The cross-sectional area of the root at the maximum sinus bulge was used to calculate the circumferential strain of the root. This was computed as (Amax−Amin)/Amin, where Amax and Amin represent the maximum and minimum areas, respectively, during the cardiac cycle.

### Histology

#### Haematoxylin and eosin staining

A total of 5 μm thick paraffin sections of ILT were washed in tap water, stained with Mayer’s haematoxylin for 5 min followed by tap water wash, slides were then incubated with 1% Eosin stain for 5 min. Rinsed briefly with tap water and dehydrated rapidly through graded methanol cleared in Xylene and mounted in DPX.

#### Alcian Blue/Sirius red staining for extracellular matrix

5 μm thick paraffin sections were washed in distilled water, stained with Weigerts Haematoxylin for 10 mins. followed by tap water wash and distilled water wash, sections were then incubated with 3% Alcian blue for 20 min followed by 1% molybdophosphoric Acid for 20 mins. After a brief distilled water wash, sections were stained with Sirius Red / Picric Acid for 60 min. Stained slides were washed briefly in distilled water, dehydrated rapidly through graded methanol cleared in Xylene and mounted in DPX.

#### Elastic Van Gieson staining

5 μm thick paraffin sections were washed in distilled water, rinsed in 100% methanol, immersed in Miller’s elastin stain (VWR) for 2 h at room temperature, differentiated in 90% methanol for 10–20 s, washed well in tap water followed by distilled water, immersed in Van Giesson stain for 5 min, rinsed briefly in distilled water, dehydrated rapidly through graded methanol cleared in Xylene and mounted in DPX.

#### Immunochemistry

Valves were explanted at 1 week, 2weeks, 9 weeks, 13 weeks, and 26 weeks and were washed with PBS, fixed in 4% paraformaldehyde for 24 h. The anterior root, left posterior, and right posterior root were dissected out and washed in phosphate buffered saline (PBS). Specimens were then immersed in 10% formol saline and processed for paraffin blocks. 5 µm thick paraffin sections were cut and dewaxed and rehydrated to water. Antigen retravel was carried out in 0.1 M citrate buffer (pH 6) and microwaved for 10 min at high power, leave for further 20 min to cool down before blocking for endogenous peroxidases using 0.3% hydrogen peroxide in PBS. Sections were washed twice in PBS and blocked using 10% normal horse serum in 2% bovine serum albumin (w/v) (BSA) in PBS containing 1% v/v Tween-20 followed by incubation with primary antibodies listed in the Supplementary Table 1, overnight in the fridge. Primary antibodies were then removed by washing the sections 3 times in PBS followed Vectastain (R) Elite ABC-HRP kit, peroxidase RTU (universal kit) Reactivity was detected using diaminobenzidine tetrahydrochloride (DAB tablets- Sigma) (25 mg/ml) and hydrogen peroxide (0.01% w/v). Sections were then counter stained with Mayers haematoxylin and stained slides were scanned using Hamamatsu Nanozoomer.

#### Immunofluorescence staining

Prior to immunofluorescence staining, 5 µm thick paraffin sections of sheep explants were dewaxed and rehydrated to water. Antigen retrieval was carried out by microwaving the slides in 0.1 M citrate buffer. To reduce non-specific binding, the slides were incubated with 10% normal goat serum in 2% bovine serum albumin (BSA -Sigma) for 40 min. Specimens were then incubated overnight in a moist chamber with a cocktail of antibodies consisting smooth muscle alpha actin (mouse from DAKO) and CD31 (rabbit- Abcam) Negative controls consisted of 10% normal goat serum in 2% BSA in PBS. After thorough washing, all the specimens were incubated with secondary antibodies, goat anti rabbit Alexa Fluor 634 for rabbit polyclonals and goat anti mouse Alexa Fluor 488 (Life Technologies) for 1 h at 1:1000 dilution. After washing several times with PBS sections were incubated with DAPI (1:5000) (sigma) for 15 min and to reduce autofluorecence, sections were incubated with Vector TrueView quenching kit (Vector laboratories) for 3 min and mounted using Permafluor (Beckman Coulter). Stained sections were imaged using a Zeiss LSM 880 confocal microscope.

#### Silver staining

Silver Staining for nerves was carried out according to Bielschowsky’s silver stain method as described in Theory and practice of histological techniques by Bancroft and Stevens^60^.

#### Transmission electron microscopy

Pieces of tissue (2 mm^2^) were excised from different regions of the remodelled valve and fixed in 3% glutaraldehyde (Agar Scientific Ltd., Essex, UK) in 0.1 M cacodylate buffer for 2 h. After two buffer washes, the secondary fixation with 1% osmium tetroxide (Agar Scientific Ltd.) in 0.1 M cacodylate buffer was carried out for 1 h at room temperature. The specimen was then dehydrated through an increasing ethanol series, starting from 25% ethanol. After dehydration, the tissue was transferred to propylene oxide (Sigma-Aldrich, UK). The tissue was then infiltrated with 1:1 propylene oxide:Araldite CY212 resin overnight. After two changes of fresh resin, for a minimum of 3 h each, the tissue was embedded in Araldite CY212 resin and polymerized at 60 °C for 18 h. Ultra-thin sections were cut using a Diatome diamond knife on a Reichert-Jung Ultracut E ultramicrotome, floated onto distilled water, collected on copper grids, and stained with 2% uranyl acetate and lead citrate for 10 min in each solution. The stained sections were viewed on JEOL 1200 electron microscope and digital images were taken using a Gatan camera.

#### Tissue processing for Scanning Electron Microscopy

Pieces of valve leaflet and sinus wall specimens were fixed in 2.5% glutaraldehyde in 0.1 M sodium cacodylate buffer for 2 h at room temperature. After 2 buffer washes, the secondary fixation with 1% osmium tetroxide in 0.1 M cacodylate buffer was carried out at room temperature for 1 h. Specimens were dehydrated in ascending ethanol series starting from 20% ethanol. Then the specimens were treated with hexamethyl disilazane (HMDS- Sigma) three times 2 min in each solution, air-dried, and mounted on SEM stubs. The stubs were sputter coated with gold and viewed in JEOL JSM-5500 LV SEM (JEOL UK Ltd). All chemicals were obtained from Agar Scientific Ltd, Stanstead, Essex, UK.

#### Energy dispersive X-ray spectroscopy

As one of the 9-week explants showed a calcific nodule, elemental analysis by energy-dispersive X-ray spectroscopy was carried out on a dewaxed and rehydrated 10 μm thick paraffin wax section, air-dried and mounted on SEM stubs coated with Gold/Palladium using a JEOL 6010 analytical scanning electron microscope.

#### Gel permeation chromatography

Sample (5 m × 5 mm size) of tissue/scaffold composite were submerged with 5% NaOCl (sigma) for 1 h to remove biological tissue, followed by 3 times wash in dH_2_O, PCL component was dissolved in 2 ml of chloroform (Sigma), followed by air drying and redissolved with THF (VWR) at 1 mg/ml PCL/THF solution. Samples were pre-filtered with PTFE filter at 0.2 µm prior to analysis. Sample were analysed by Agilent 1260 infinity II GPC/ size exclusion chromatography system with flow rate 1 ml/min at 25 °C. Weight-averaged molecular weights (Mw) of the PCL were calculated using polystyrene standard (Agilent infinity lab EasiVial) standard curve (*M*_n, EXP_ = *X* × *M*_n, SEC_) with correcting coefficients X = 0.6 for PCL as determined previously^61^.

#### Statistics and reproducibility

All Statistical comparisons were performed using GraphPad Prism v10.0.1.All data was tested for normality, unless otherwise stated. In vitro hydrodynamic testing analysis: Statistical comparisons between the control group (Freestyle Medtronic valve) and the experimental group (HCCV) were conducted using a two tailed t-test, with a sample size of *n* = 4, independent samples for each group. A *p*-value of less than 0.05 was considered statistically significant for the analysis.

Strain analysis: Statistical comparisons were conducted between the HCCV group and the control group (native sheep) using a two-tailed t-test, assuming unequal variance. The sample size was *n* = 5 (biologically independent samples) for the HCCV group and *n* = 3 (biologically independent samples) for the control group. A *p*-value of less than 0.05 was considered statistically significant.

HCCV morphometry (*n* = 4, biologically independent samples) and GPC analysis (*n* = 3, biologically independent samples): Statistical comparisons were performed using one way ANOVA analysis followed by post-hoc Tukey’s multiple comparison test. HCCV leaflet thickness pre and post-implantation was compared using a two-tailed Mann Whitney test and a *p*-value of less than 0.05 was considered statistically significant.

### Reporting summary

Further information on research design is available in the Nature Portfolio Reporting Summary linked to this article.

## Supplementary information

Supplementary figures and table

Description of Supplementary Materials

Supplementary video 1

Supplementary video 2

Supplementary video 3

Supplementary data

reporting summary

## Data Availability

Source data is available in the supplementary data.

## Code availability

The custom Matlab code for GOA analysis is freely available via Github under General public licence. The code is available at 10.5281/zenodo.8275999.
