# Author Correction: Valvulogenesis of a living, innervated pulmonary root induced by an acellular scaffold

**DOI:** 10.1038/s42003-025-08927-7

**Published:** 2025-10-13

**Authors:** Magdi H. Yacoub, Yuan-Tsan Tseng, Jolanda Kluin, Annemijn Vis, Ulrich Stock, Hassiba Smail, Padmini Sarathchandra, Elena Aikawa, Hussam El-Nashar, Adrian H. Chester, Nairouz Shehata, Mohamed Nagy, Amr El-sawy, Wei Li, Gaetano Burriesci, Jacob Salmonsmith, Soha Romeih, Najma Latif

**Affiliations:** 1 Magdi Yacoub Institute, Harefield, UK; 2 National Heart and Lung Institute, Imperial College London, London, UK; 3 Aswan Heart Science Center, Magdi Yacoub Foundation, Aswan, Egypt; 4 Department of Cardiothoracic Surgery, Erasmus MC, Rotterdam, The Netherlands; 5 Amsterdam UMC, University of Amsterdam, Department of Cardiothoracic Surgery, Amsterdam, The Netherlands; 6 Royal Brompton and Harefield Hospital, London, UK; 7 Cardiovascular Medicine, Brigham and Women’s Hospital, Harvard Medical School, Boston, USA; 8 Department of Bioengineering, Imperial College London, London, UK; 9 Department of Computing, Imperial College London, London, UK; 10 Cardiovascular Engineering Laboratory, UCL Mechanical Engineering, University College London, London, UK; 11 Bioengineering Unit, Ri.MED Foundation, Palermo, Italy

Correction to: *Communications Biology* 10.1038/s42003-023-05383-z, published online 07 October 2023

In the version of the article initially published, the image shown in the leftmost panel of Fig. 2f was an inadvertent mirrored duplicate of the leftmost image in Fig. 2h; the image in Fig. 2f has now been replaced in the HTML and PDF versions of the article.

