# Supplementary material for: Valvulogenesis of a living, innervated pulmonary root induced by an acellular scaffold
Source: Commun Biol. 2023 Oct 7;6:1017. doi: 10.1038/s42003-023-05383-z (PMC10560219; doi:10.1038/s42003-023-05383-z)
Supplement: Supplementary file 1 — Supplementary figures and table [file 42003_2023_5383_MOESM1_ESM.pdf]

Supplementary Figure 1.
Schematic showing the number of animals, the time points and failure modes of HCCV used in the study.

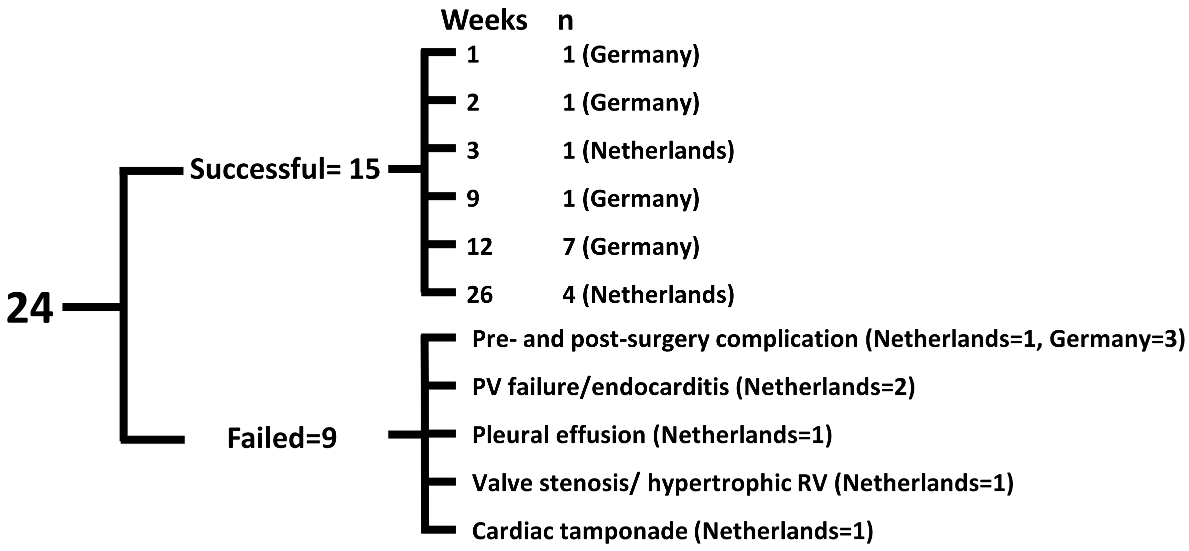

Schematic showing the breakdown of the number of animals, the time points of explantation, reasons for failure and country of implantation.

Supplementary Figure 2.
Stained sections of a normal sheep pulmonary valve (PV) sinus to that of HCCV sinus .

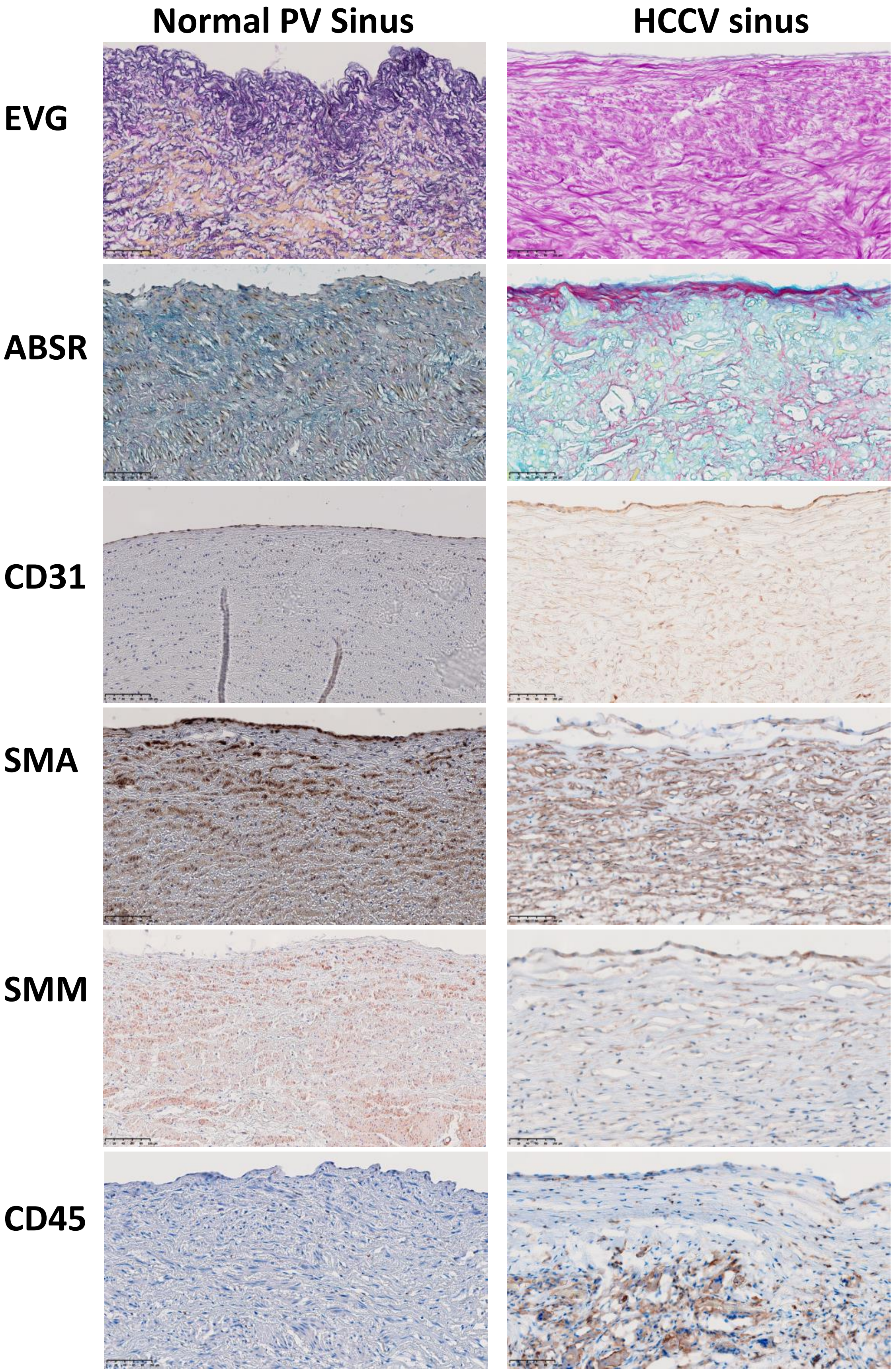

Supplementary Figure 3.
Images of calcification and elemental analysis.

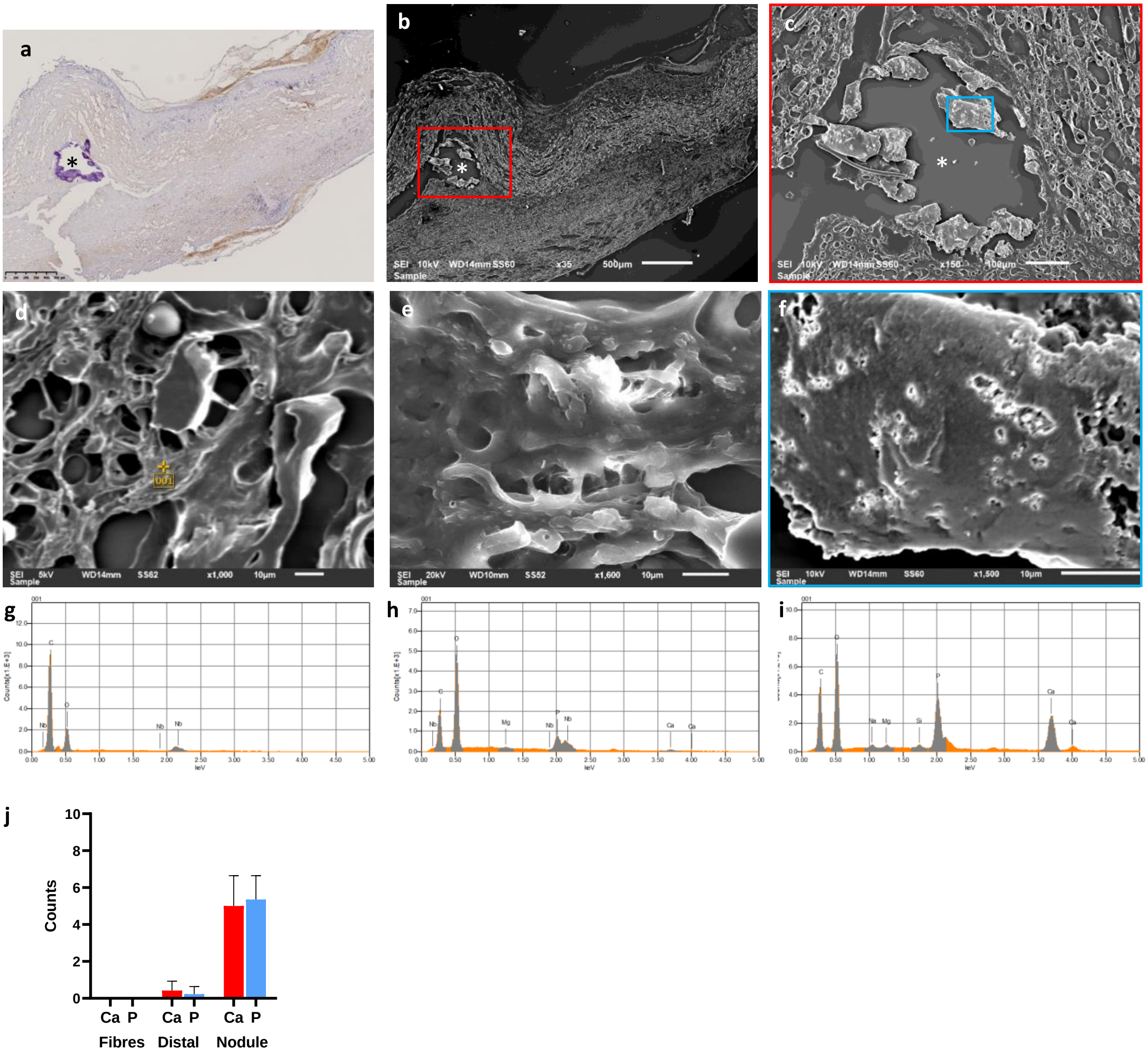

One cusp showed one nodule of calcification (*) in the middle of one leaflet (a-c, f). Light microscopy image of nodule (a), SEM of same nodule (b) with magnified image (c) and high magnification of calcified material (f). SEM of nanofibers away (d) and close to nodule (e). Representative energy dispersive X-ray analysis of region in panel d showed no signals for calcium or phosphorus in the nanofibrous area (g), of region in panel E showed mean counts of 0.42 ± SD 0.51 and counts of 0.233 ± SD 0.40 for calcium (3.69 KeV) and phosphorus (2.01 KeV) respectively (h) and region in panel f showed mean counts of 5.00 ± SD 1.28 and 5.35 ± 1.28 for calcium and phosphorus within the nodule (i). Quantification of the amount of calcium (Ca) and phosphorus (P) in the nanofibers (Fibers) in the sinus wall, distal to the nodule in the leaflet (Distal) and the calcified nodule (Nodule) (j). Scale bars are on each panel (a-f).

Supplementary Figure 4. Echo *In vivo* function

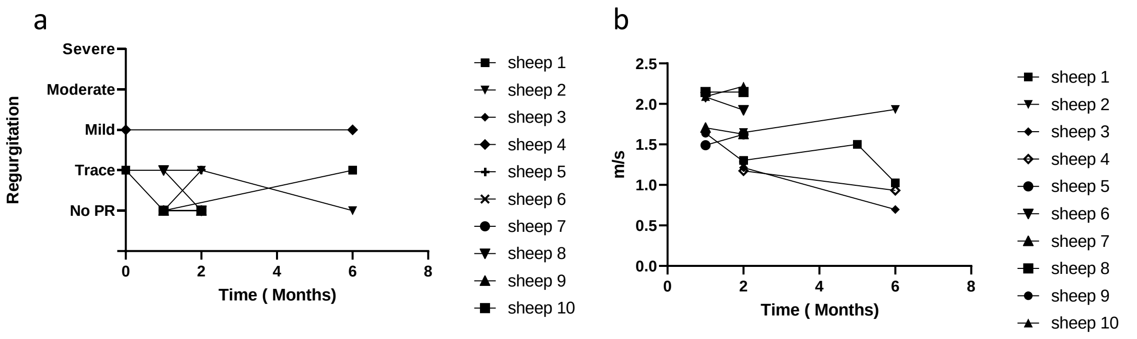

The pulmonary regurgitation in sheep implanted with individual HCCV was qualitatively assessed using echo, as shown in Supplementary Figure 4a. Figure 4b shows the normalised Doppler Vmax of the individual HCCV-implanted sheep was assessed, and there were no significant signs of valvular stenosis up to 6 months.

Supplementary Table 1. Antibody information

Antibody	Host species	Dilution	Pre treatment	Company
Collagen I	Rabbit	1:100	0.25% hyaluronidase, 10 min at RT	Abcam-34710
Collagen III	Rabbit	1:200	0.25% hyaluronidase, 10 min at RT	Rockland-#600-401-105
Fibronectin	Mouse	1:100	0.1 M citrate buffer antigen retrieval	Acris-AM50112PU-T
Chondroitin sulphate	Mouse	1:300	Chondroitinase ABC 25U/ml, 30 min at 37 degrees	Sigma-C-8035
Heparan sulphate	Mouse	1:200	Chondroitinase ABC 25U/ml, 30 min at 37 degrees	Seikaku-370255
**VICS**				
α-SMA	Mouse	1:200	0.1 M citrate buffer antigen retrieval	Dako-M0851
vimentin	Rabbit	1:100	0.1 M citrate buffer antigen retrieval	cell signalling-#5741
**VECs**				
CD31	Rabbit	1:800	0.1 M citrate buffer antigen retrieval	Abcam- ab134168
CD34	Mouse	1:6000	0.1 M citrate buffer antigen retrieval	Abcam-ab81289
vWF	Rabbit	1:1200	0.1 M citrate buffer antigen retrieval	DAKO-A0082
eNOS	Mouse	1:200	0.1 M citrate buffer antigen retrieval	BD bioscience-610296
**Smooth Muscle Cell**				
SMM	Rabbit	1:100	0.1 M citrate buffer antigen retrieval	Abcam-ab133567
**Immunological Markers**				
CD45	Rabbit	1:1000	0.1 M citrate buffer antigen retrieval	Biorad-MCA2220GA
CD68	Mouse	1:500	0.1 M citrate buffer antigen retrieval	Abcam-ab13363
CD163	Mouse	1:5000	0.1 M citrate buffer antigen retrieval	Invitrogen-MA1-82342
CCR7	Rabbit	1:300	0.1 M citrate buffer antigen retrieval	Abcam 227768
CD14	Mouse	1:50	0.1 M citrate buffer antigen retrieval	Biolegend 301802
**Adipogenic Markers**				
FABP4	Rabbit	1:4000	0.1 M citrate buffer antigen retrieval	Abcam-ab 92501
Leptin	Rabbit	1:8000	0.1 M citrate buffer antigen retrieval	Novus biologicals-NB 300-611
PPARγ	Rabbit	1:10000	0.1 M citrate buffer antigen retrieval	Proteintech-16643-1
**Neuronal Markers**				
Neurofilament protein	Mouse	1:100	0.1 M citrate buffer antigen retrieval	Dako-2F11
Tyrosine hydroxylase	Mouse	1:100	0.1 M citrate buffer antigen retrieval	Thermofisher- MAB318
Neuropeptide Y	Rabbit	1:2500	0.1 M citrate buffer antigen retrieval	Abcam- ab30914
Vasoactive Intestinal Polypeptide	Rabbit	1:800	0.1 M citrate buffer antigen retrieval	Abcam-ab 22736
