# Supplementary material for: Valvulogenesis of a living, innervated pulmonary root induced by an acellular scaffold
Source: Commun Biol. 2023 Oct 7;6:1017. doi: 10.1038/s42003-023-05383-z (PMC10560219; doi:10.1038/s42003-023-05383-z)
Supplement: Supplementary file 2 — Description of Supplementary Materials [file 42003_2023_5383_MOESM2_ESM.docx]

**Description of Additional Supplementary Files**

**File name:** Supplementary video 1

**Description:** HCCV showing functionality immediately after implantation.

**File name:** Supplementary video 2

**Description:** High speed video of HCCV.

**File name:** Supplementary video 3

**Description:** High speed video of Medtronic Freestyle valve.

**File name:** Supplementary Data

**Description:**

Data: Tab Figure 1q: The source data behind the graphs in Figure 1q.

Data: Tab Figure 1r: The source data behind the graphs in Figure 1r.

Data: Tab Figure 1s: The source data behind the graphs in Figure 1s.

Data: Tab Figure 2b: The source data behind the graphs in Figure 2b.

Data: Tab Figure 2c: The source data behind the graphs in Figure 2c.

Data: Tab Figure 1q: The source data behind the graphs in Figure 1q.

Data: Tab leaflet thickness: The source data behind leaflet thickness measurement.

Data: Tab Figure 6o: The source data behind the graphs in Figure 6o.

Data: Tab Figure 6r: The source data behind the graphs in Figure 6r.
